# Advertisement calls and DNA sequences reveal a new species of *Scinax* (Anura: Hylidae) on the Pacific lowlands of Ecuador

**DOI:** 10.1371/journal.pone.0203169

**Published:** 2018-09-26

**Authors:** Santiago R. Ron, William E. Duellman, Marcel A. Caminer, Diana Pazmiño

**Affiliations:** 1 Museo de Zoología, Escuela de Biología, Pontificia Universidad Católica del Ecuador, Quito, Ecuador; 2 Biodiversity Institute, University of Kansas, Lawrence, Kansas, United States of America; 3 Centre for Sustainable Tropical Fisheries and Aquaculture, College of Science and Engineering, James Cook University, Townsville, QLD, Australia; University of Arkansas, UNITED STATES

## Abstract

*Scinax* is a speciose genus of Neotropical hylid frogs. We describe a new species from western Ecuador (elevations between 0 and 1207 m) using morphology, vocalizations, and DNA sequences. We also present a new phylogeny for *Scinax* based on mitochondrial DNA genes 12S rRNA, Cytochrome Oxidase sub-unit I, Cytochrome B, 16S rRNA, NADH dehydrogenase subunit 1, and adjacent tRNAs. The new species, *Scinax tsachila* sp. nov. was previously confused with *S*. *quinquefasciatus*, a morphologically similar sympatric species. They differ by having markedly different advisement calls, distinct skin texture in the dorsum, and different bone coloration. The new species is sister to *S*. *elaeochroa*, a species that differs in advertisement call and color pattern. We provide an updated species account for *Scinax quinquefasciatus* and a redescription of its holotype.

## Introduction

Genetic data have revealed the existence of many species of frogs that show little morphological differences. This is especially true among Andean strabomantid frogs of the genus *Pristimantis* as shown by Duellman and Hedges [1,2], as well as in Amazonian hylids, where Caminer and Ron [3] showed that “*Boana fasciata*” contains up to 11 species. Likewise, genetic data revealed that the hemiphractid frog *Gastrotheca monticola* in northern Andean Peru was made up of three species that were genetically distinct but morphologically alike [4]. Other genetic analyses have corroborated the existence of cryptic species in four families in northwestern South America—Centrolenidae: *Nymphargus* [5]; Strabomantidae: *Pristimantis* [6]; Dendrobatidae: *Hyloxalus* [7]; Hylidae: *Boana* [3], *Dendropsophus* [8], *Osteocephalus* [9,10]; and Leptodactylidae: *Engystomops* [11,12].

An additional genus in which cryptic diversity has been documented is *Scinax* [13,14]. *Scinax* has 118 species [15] and its monophyly is well supported (e.g., [16]). Based on genetic data, Fouquet et al. [13] reported the existence of up to six undescribed species within *Scinax ruber*. More recently, Ferrão et al. [14] reported that 82% of regional species richness of *Scinax* is still undescribed in two areas in Amazonian Brazil. Nevertheless, since Duellman and Wiens [17], only one species has been described from western South America, *Scinax iquitorum*, a member of the *Scinax ruber* Group [18]. In the late 1960s personnel from the University of Kansas undertook fieldwork in the Pacific lowlands of Ecuador. They collected many specimens of a medium-sized frog that they referred to *Hyla quinquefasciata* Fowler, a species that was included in the “*Hyla rubra* Group” (*auctorum*). During the following decades many more specimens were collected and identified as *Hyla quinquefasciata*, which had been the only species of the *S*. *ruber* group known from the Chocoan Region. Recent morphologic and genetic analyses of those specimens have shown the presence of two cryptic species within “*Scinax quinquefasciatus*”. Herein we name the new cryptic species and provide an updated species account for *Scinax quinquefasciatus*.

## Methods

### Ethics statement

Voucher specimens and tissue samples were obtained following ethical and technical protocols [19]. Vouchers were euthanized with commercial roxicaine (anesthetic spray), fixed in 10% neutral-buffered formalin and preserved in 70% ethanol. Field permits were issued by the Ecuadorian Ministry of Environment (001–10 IC-FAU-DNB/MA, 002-16-IC-FAU-DNB/MA, 003–15 IC-FAU-DNB/MA, 005-12-IC-FAU-DNB/MA, 005–14 IC-FAU-DNB/MA, 008–09 IC-FAU-DNB/MA). This study was evaluated and approved by the DGA (Dirección General Académica) of the Pontificia Universidad Católica del Ecuador in accordance with the guidelines for environmental and social impacts of research projects. The Dirección General Académica committee individually evaluates each project to determine its observance of its norms for ethical scientific research. Genetic data were obtained under Genetic Resources Access Contract No MAE-DNB-CM-2015-0025 issued by Ministerio de Ambiente del Ecuador to Pontificia Universidad Católica del Ecuador.

### Morphology

Examined specimens are deposited in the following collections: Academy of Natural Sciences of Drexel University, USA (ANSP); Biodiversity Institute, University of Kansas, Lawrence, USA (KU); Museo de Zoología, Pontificia Universidad Católica del Ecuador, Quito, Ecuador (QCAZ). Sex was determined by the presence of a vocal sac or ovarian eggs. Character terminology follows Duellman [20]. Taxonomy follows AmphibiaWeb [15]. Webbing formula follows Myers and Duellman [21].

We measured seven morphometric variables: snout–vent length (SVL), head width (HW), head length (HL), tibia length (TL), foot length (FL), diameter of eye (ED), and diameter of tympanum (TD). In the description of the holotype of the new species we also measured the width of the upper eyelid, interorbital distance, internarial distance, and eye-nostril distance. All measurements were taken in the manner described by Duellman [20,22]; numbered diagnosis follows Duellman and Wiens [17]. Specimens measured for *S*. *tsachila* sp. nov. were QCAZ 3511–12, 15541, 23175, 23183–85, 23672–73, 23678, 26104, 27017, 30764, 31757, 34101, 39880, 40843, and 58652; specimens measured for *S*. *quinquefasciatus* were QCAZ 12625, 12803, 15005, 19926, 23179, 23383–85, 23398, 23451–56, 23468, 23538, 23593, 23660, 23689, 26801, 26940, 27018, 30596, 39867–68, 42228, and 42254. Measurements were made using digital calipers (to the nearest 0.1 mm). Specimens examined are listed in S1 Appendix. Discriminant Function Analysis (DFA) was used to assess the degree of morphometric differentiation between adult males of the new species and *S*. *quinquefasciatus*. Only well preserved specimens [23] were measured for the following six morphological variables: snout–vent length (SVL), head width (HW), head length (HL), tibia length (TL), foot length (FL), and diameter of tympanum (TD). We applied the DFA to the raw variables, without size correction, because we wanted to assess discriminability among species based on all the data, including SVL. Sample sizes for the DFA are *S*. *quinquefasciatus* 24 males and *S*. *tsachila* sp. nov. 12 males. The DFA was conducted in JMP® 9.01 [24].

### Phylogenetic analyses and genetic distances

DNA was extracted from muscle or liver tissue preserved in 95% ethanol or tissue storage buffer, using standard phenol–chloroform extraction protocols ([25]). We used a polymerase chain reaction (PCR) to amplify DNA fragments for mitochondrial genes 12S rRNA (12S), Cytochrome Oxidase sub-unit I (COI), Cytochrome B (CytB), two overlapping fragments for the last ~320 bp of 16S rRNA (16S), NADH dehydrogenase subunit 1 (ND1) and adjacent tRNAs (tRNA^Leu^, tRNA^Ile^ and tRNA^Gln^) using the primers listed in Folmer et al. [26], Goebel et al. [27], Heinicke et al. [28], Moen and Wiens [29], Fouquet et al. [30], and Wiens et al. [31]. PCR amplification was performed under standard protocols and sequenced by the Macrogen Sequencing Team (Macrogen Inc., Seoul, Korea). The combined DNA matrix had up to 5447 bp. Percentage of missing data in the matrix was 69.7%.

The newly generated DNA sequences are available in GenBank under accession numbers listed in Table 1. We also included sequences of available species of *Scinax* at GenBank until June 2015. Those sequences were originally published by Bell et al. [32], Brusquetti et al. [33], Carnaval [34], Darst and Cannatella [35], Faivovich et al. [16,36], Fouquet et al. [13,37,38], Frost et al. [39], Jansen et al. [40], Jungfer et al. [10], Moen and Wiens [29], Salducci et al. [41,42], Schulze et al. [43] and Wiens et al. [31,44]. We also included samples of *Dendropsophus*, *Dryaderces*, *Boana*, *Osteocephalus*, and *Trachycephalus* as outgroups. Preliminary sequence alignment was done with MAFFT 7.2 software with the L-INS-i algorithm [45]. Protein-coding genes were colored according to amino acids in MESQUITE (version 3.01; [46]) and all sequences in the matrix were visually examined and the alignment was manually corrected if needed[46]. The aligned matrix is available at https://zenodo.org under DOI 10.5281/zenodo.1317007. The matrix was partitioned to allow independent inferences of models of evolution by gene and by codon position in coding genes. We used PARTITIONFINDER v. 1.1.1 [47] to estimate simultaneously both the best-fit model for each partition and the best partition strategy for our data.

**Table 1:** List of specimens included in the phylogenetic analysis and their GenBank accession numbers.

Museum No.	Species	Genbank Accession No.				
		*12S*	*16S*	*ND1*	*COI*	*CytB*
QCAZ 39444	*Scinax funereus*	MH662465	MH662480	MH662531	—	—
QCAZ 43799	*S*. *funereus*	MH662466	MH662481	MH662533	—	—
QCAZ 51043	*S*. *funereus*	MH662467	MH662504	MH662536	—	—
QCAZ 43755	*S*. *garbei*	MH662463	MH662501	MH662537	—	—
QCAZ 46403	*S*. *garbei*	MH662470	MH662482	—	—	—
QCAZ 48844	*S*. *garbei*	MH662471	MH662483	MH662532	—	—
QCAZ 23398	*S*. *quinquefasciatus*	MH662457	MH662493	MH662539	—	MH662526
QCAZ 23451	*S*. *quinquefasciatus*	MH662472	MH662494	MH662540	MH662506	—
QCAZ 23539	*S*. *quinquefasciatus*	MH662458	MH662495	MH662541	MH662505	MH662527
QCAZ 26940	*S*. *quinquefasciatus*	MH662459	MH662497	MH662538	MH662507	MH662528
QCAZ 43729	*S*. *ruber*	MH662468	MH662500	MH662535	—	—
QCAZ 51062	*S*. *ruber*	MH662469	MH662477	MH662530	—	—
QCAZ 43681	*S*. sp.	MH662464	MH662499	MH662534	—	—
KU 218492	*S*. *tsachila* sp. nov	MH662446	—	—	MH662508	—
KU 218493	*S*. *tsachila* sp. nov	MH662447	MH662484	MH662543	MH662509	—
KU 218494	*S*. *tsachila* sp. nov	MH662448	MH662485	MH662544	MH662510	—
KU 218495	*S*. *tsachila* sp. nov	MH662449	MH662486	MH662545	MH662511	—
KU 218498	*S*. *tsachila* sp. nov	MH662450	MH662487	MH662546	MH662512	—
KU 218503	*S*. *tsachila* sp. nov	MH662451	MH662488	MH662547	MH662513	—
KU 218504	*S*. *tsachila* sp. nov	MH662460	MH662489	MH662553	MH662514	—
KU 218505	*S*. *tsachila* sp. nov	MH662452	MH662490	MH662554	MH662515	—
QCAZ 23183	*S*. *tsachila* sp. nov	MH662453	MH662491	MH662548	MH662516	—
QCAZ 23184	*S*. *tsachila* sp. nov	MH662454	MH662492	MH662542	MH662517	—
QCAZ 23185	*S*. *tsachila* sp. nov	MH662455	MH662478	MH662555	MH662518	—
QCAZ 23672	*S*. *tsachila* sp. nov	MH662462	MH662496	MH662529	MH662519	—
QCAZ 23673	*S*. *tsachila* sp. nov	MH662474	—	—	MH662520	—
QCAZ 23678	*S*. *tsachila* sp. nov	MH662461	—	—	MH662521	—
QCAZ 39880	*S*. *tsachila* sp. nov	MH662473	MH662498	MH662549	MH662522	—
QCAZ 40843	*S*. *tsachila* sp. nov	MH662475	MH662479	MH662550	MH662523	—
QCAZ 45423	*S*. *tsachila* sp. nov	MH662456	MH662502	MH662551	MH662524	—
QCAZ 45424	*S*. *tsachila* sp. nov	MH662476	MH662503	MH662552	MH662525	—

Phylogenetic trees were obtained using maximum likelihood searches with software GARLI 2.0 [48]. We made 20 independent searches, 10 starting from random trees and 10 from stepwise addition trees. We modified the settings for the number of generations without topology improvement required for termination (genthreshfortopoterm = 200000) to increase thoroughness of the search of the tree space. Other settings were set on default values. We evaluated the exhaustiveness of the global search by comparing the final maximum likelihood value among replicate searches. We considered that the searches were effective in finding the best trees when more than 50% of the replicates had maximum likelihood values within 2 units of the best global search. Node support was assessed with 200 pseudoreplicate non-parametric bootstraps, using the same settings of the full search but with one replicate per run.

We calculated genetic distances as tentative evidence of the distinctiveness of the new species. We obtained sequences of mitochondrial 16S gene for *S*. *tsachila* sp. nov. specimens QCAZ 39880, 45423, and 65690. Those sequences were compared with homologous GenBank sequences for *S*. *elaeochroa*: MVZ203919 from Heredia, Costa Rica (AY843757) and MVZ149785 from Cahuita, Limón, Costa Rica (EF376076). Uncorrected *p*-genetic distances were calculated with software MEGA v.7.0 [49]. Standard errors were estimated under MEGA bootstrap option.

### Advertisement calls

Advertisement call recordings were made with a Sennheiser™ ME-67 directional microphone and Sony^TM^ WM-D6C analog tape recorder. Calls of *Scinax elaeochroa* were also obtained from the audio archive of the Macaulay Library at the Cornell Lab of Ornithology (http://macaulaylibrary.org/). Calls were analyzed using Raven 1.5 (www.birds.cornell.edu/raven) at a sampling frequency of 48.0 kHz and a frequency resolution of 11.7 Hz. Measured call variables were: (1) call rate: number of calls per second, (2) dominant frequency: frequency with the most energy, measured along the entire call, (3) fundamental frequency: frequency with the greatest amount of sound energy in the first harmonic, measured along the entire call, (4) call duration: time from the beginning to the end of the call, (5) number of pulses: number of pulses in the call, (6) pulse rate: number of pulses/call duration, (7) rise time: time from the beginning of the call to the point of its maximum amplitude. Several calls or notes were analyzed per individual to calculate an individual average. All variables were used in a Principal Components Analysis (PCA) to assess the degree of acoustic differentiation between calls from seven males of *S*. *elaeochroa* (from La Lola, Palmar Sur, Puerto Viejo, and Turrialba in Costa Rica), three males of *S*. *quinquefasciatus* (from Las Palmas-Balsas, Pedernales and Rocafuerte in Ecuador), and five males of *S*. *tsachila* sp. nov. (from Arenillas, Estación Biológica Bilsa, Río Palenque, and Santo Domingo in Ecuador). Original recordings are deposited in the audio archive of the QCAZ and are available through the Anfibios del Ecuador website (https://bioweb.bio/faunaweb/amphibiaweb).

### Nomenclatural acts

The electronic edition of this article conforms to the requirements of the amended International Code of Zoological Nomenclature, and hence the new names contained herein are available under that Code from the electronic edition of this article. This published work and the nomenclatural acts it contains have been registered in ZooBank, the online registration system for the ICZN. The ZooBank LSIDs (Life Science Identifiers) can be resolved and the associated information viewed through any standard web browser by appending the LSID to the prefix “http://zoobank.org/”. The LSID for this publication is: urn:lsid:zoobank.org:pub:0EB37032-3F15-4816-A389-87EE0E1E3E79. The electronic edition of this work was published in a journal with an ISSN, and has been archived and is available from the following digital repositories: PubMed Central, LOCKSS.

## Results

### Phylogeny

**Fig 1:**
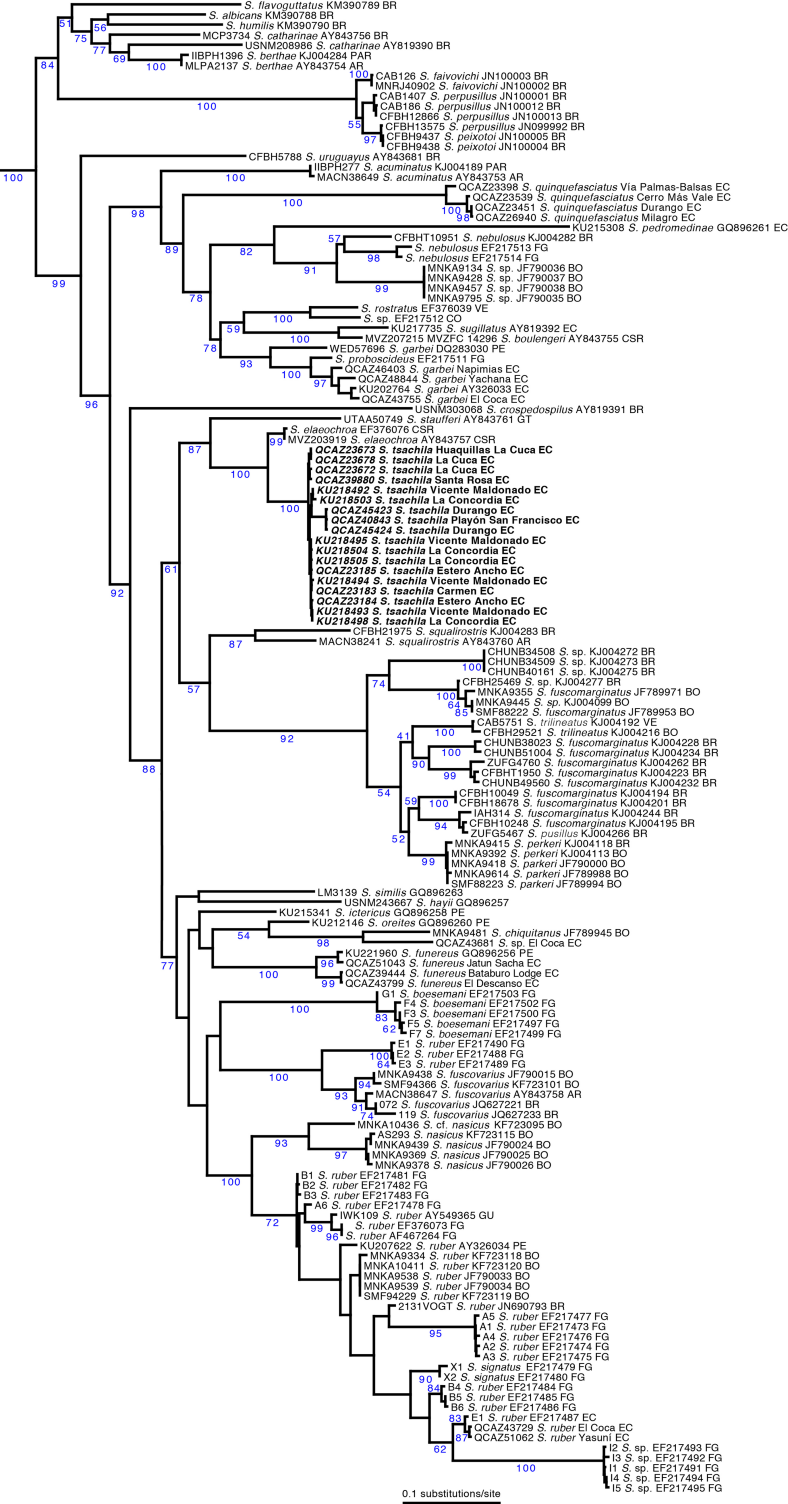
Maximum likelihood phylogram of *Scinax*. The phylogram was obtained from analysis of 5447 bp of mitochondrial DNA (gene fragments 12S, 16S, ND1, CO1, CytB, *tRNA* ^*Leu*^, *tRNA* ^*Ile*^, *tRNA* ^*Gln*^). Voucher number is shown to the left of the species name of each sample; GenBank accession number, for the 16S fragment, is shown to the right and country abbreviation at the end. Non-parametric bootstrap support values, from 200 pseudoreplicates, are shown in blue. Outgroups are not shown. Abbreviations: AR = Argentina, BO = Bolivia, BR = Brazil, CO = Colombia, CSR = Costa Rica, EC = Ecuador, FG = French Guiana, GT = Guatemala, GU = Guyana, PAR = Paraguay, PE = Peru, VE = Venezuela.

Our maximum likelihood phylogeny for the genus *Scinax* shows two strongly supported clades diverging basally (Fig 1). One includes species of the *Scinax catharinae* clade sensu Faivovich [16] (= *Ololygon sensu* Duellman et al. [50]). The other clade contains all species of the *Scinax ruber* clade (= *Scinax* + *Julianus sensu* Duellman et al. [50]). Although with fewer species, the same arrangement was reported by Wiens et al. [44] and Duellman et al. [50].

Within the *S*. *catharinae* clade we found strong support for the *S*. *perpusillus* group and the *S*. *catharinae* group as defined by Faivovich et al. [16]. The *S*. *rostratus* group has strong support and is sequentially sister to *S*. *quinquefasciatus* and *S*. *acuminatus*. *Scinax garbei* is paraphyletic relative to *S*. *proboscideus*, a topology that suggests the existence of cryptic species within *S*. *garbei*.

The new species is part of the *S*. *ruber* clade and is closely related to the Central American *S*. *elaeochroa*. Uncorrected p-genetic distances (16S) between the new species and *S*. *elaeochroa* range from 3.6% (SE = 0.5) to 5.1% (SE = 1.0). These 16S distances are above the 3.0% threshold that is generally interpreted as indicative of interspecific differences in anurans (e.g., [38]). Genetic distances within *S*. *tsachila* sp. nov. range from 0.6 to 0.9%; two samples of *S*. *elaeochroa* are identical to each other (distance = 0%).

### Acoustic and morphometric comparisons

In the DFA classification, 33 out of 36 specimens were assigned to their correct species. Only two *S*. *quinquefasciatus* and one *S*. *tsachila* sp. nov. were misclassified. Both multivariate analyses indicate that *S*. *quinquefasciatus* and *S*. *tsachila* sp. nov. have low overlap in morphometric space.

**Fig 2:**
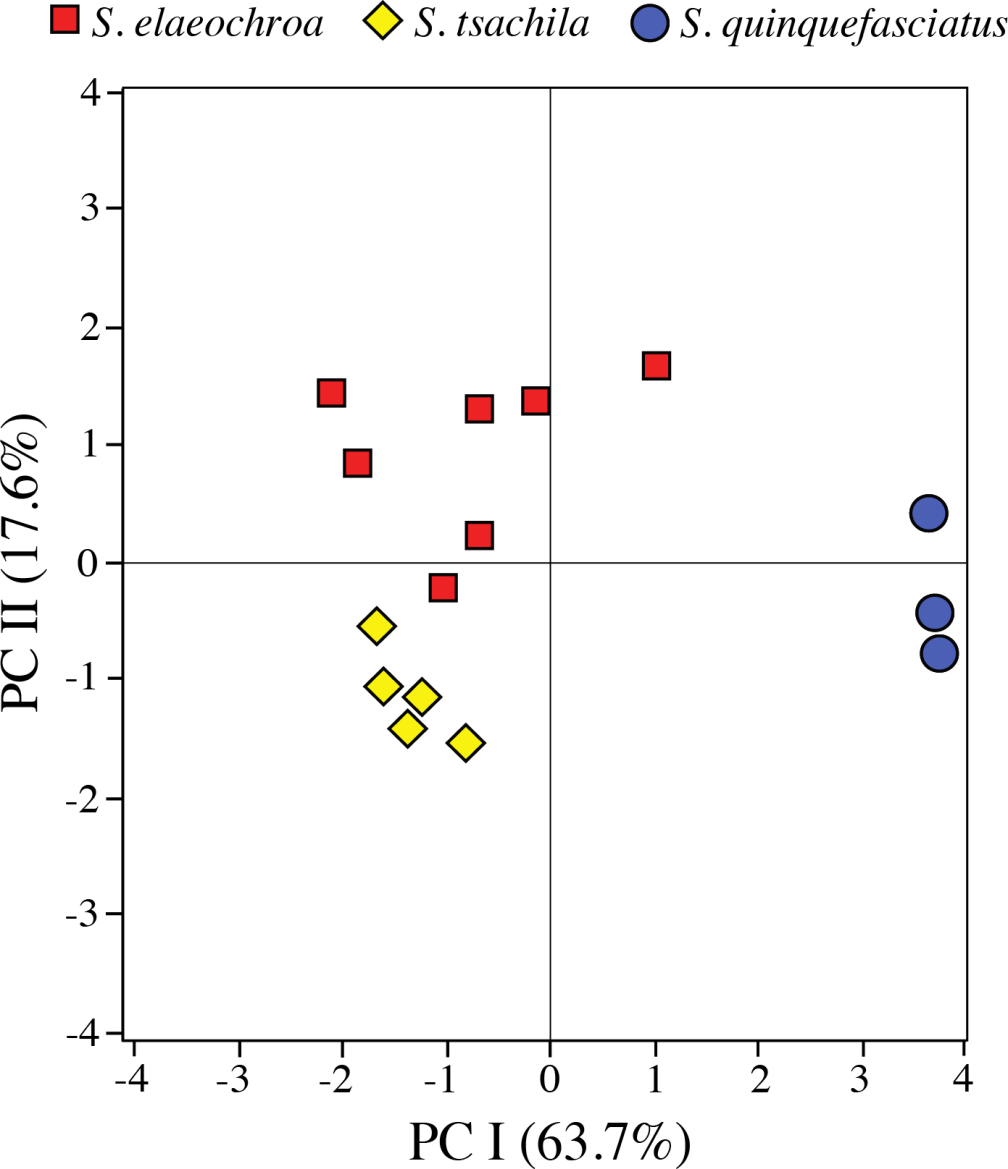
Axes I and II from principal components analysis. Based on seven acoustic variables from the advertisement calls of *Scinax elaeochroa* (7 males), *S*. *quinquefasciatus* (3), and *S*. *tsachila* sp. nov. (5). See Table 2 for character loadings on each component.

Our PCA of advertisement calls from 15 males resulted in two PCs with eigenvalues > 1.0. The two PCs combined accounted for 81.23% of the total variance. PC I (63.65% of the variance) was positively correlated with call duration, rise time and number of pulses, while PC II (17.58% of the variance) was correlated with pulse rate (Table 2). The acoustic space (as represented by PC I and PC II; Fig 2) showed significant differences among *S*. *elaeochroa*, *S*. *quinquefasciatus*, and *S*. *tsachila* sp. nov. Comparisons of PC I scores showed segregation between *S*. *elaeochroa* and *S*. *tsachila* sp. nov. relative to *S*. *quinquefasciatus*. PC II scores were significantly different between *S*. *quinquefasciatus* and *S*. *tsachila* sp. nov. compared to *S*. *elaeochroa*.

**Table 2:** Principal components analysis of calls of *Scinax*.

Variable	Character Loading	
	PC I	PC II
Call duration	**0.968**	-0.037
Rise time	**0.842**	0.134
Call fundamental frequency	-0.847	0.219
Call dominant frequency	0.798	0.425
Pulse number	**0.888**	-0.285
Pulse rate	-0.561	-**0.706**
Call rate	-0.590	0.633
Eigenvalue	4.455	1.231
%	63.651	17.588

Character loadings, eigenvalues, and percentage of explained variance for Principal Components (PC) I–II are shown. The analysis was based on seven acoustic variables from the advertisement calls of *Scinax elaeochroa*, *S*. *quinquefasciatus* and *S*. *tsachila* sp. nov. Bold figures indicate highest loadings.

## Systematic accounts

The genetic, morphologic, and bioacoustic evidence demonstrates that two species have been masked under “*Scinax quinquefasciatus*” in the Chocó Region. One is *S*. *quinquefasciatus sensu stricto*. The other is an undescribed species closely related to *S*. *elaeochroa*. The new species differs from *S*. *elaeochroa* genetically (uncorrected *p*-distance >3.5% for gene 16S), in coloration, and in advertisement call (see Diagnosis in *S*. *tsachila* sp. nov.) In the following sections we provide an updated species account for *S*. *quinquefasciatus* and describe the new species.

### *Scinax quinquefasciatus* (Fowler 1913)

*Hyla quinquefasciatus* Fowler, 1913: 160. Holotype.—ANSP 18115 from “Mountains above Chimbo, 10,000–10,800 feet elevation,” Provincia Chimborazo, Ecuador (corrected to Durán, Provincia Guayas, by Duellman [51]), collected by S. N. Rhoads on 12 February 1911.

*Ololygon quinquefasciata—*Fouquette and Delahoussaye, 1977: 392.

*Scinax quinquefasciata—*Duellman and Wiens, 1992: 23.

#### Diagnosis

(1) Average SVL in males 32.4 mm (range 27.6–38.2), in females 36.6 mm (range 33.9–38.9; Table 3); (2) snout acutely rounded in dorsal view and in profile; (3) ulnar and tarsal tubercles absent; (4) enlarged heel tubercle absent; (5) tubercles absent on lower jaw; (6) skin on dorsum smooth to shagreen with tubercles varying from scattered to abundant; (7) diameter of tympanum ~18% of head length; (8) dorsum pale brown to brown with irregular darker stripes; (9) flanks with a longitudinal dark brown band starting in the tympanum and varying in length from being restricted to the anterior half of the flank to reaching the groin; (10) posterior surfaces of thighs without markings; (11) iris cream to bronze with brown reticulations.

**Table 3:** Descriptive statistics for morphometric measurements of adult *Scinax quinquefasciatus* and *S*. *tsachila* sp. nov.

	*S*. *quinquefasciatus* (n = 24)			*S*. *tsachila* sp. nov. (n = 12)		
Males	Mean	SD	Range	Mean	SD	Range
SVL	32.4	2.5	27.6–38.2	31.6	2.2	27.2–34.2
Tibia	16.7	1.6	13.8–19.1	15.5	1.7	13–17.8
Foot	14.1	1.3	11.2–16.9	13.5	1.2	11.3–15.1
Head width	9.9	0.8	7.8–11.3	10.0	1.0	8.1–11.8
Head length	11.1	0.8	8.8–12.7	11.1	1.2	9.2–13.8
Eye diameter	3.9	0.4	3.1–4.5	4.0	0.5	3.2–4.4
Tympanum diameter	2.0	0.2	1.6–2.3	1.6	0.2	1.3–1.9
	*S*. *quinquefasciatus* (n = 4)			*S*. *tsachila* sp. nov. (n = 6)		
Females	Mean	SD	Range	Mean	SD	Range
SVL	36.6	2.1	33.9–38.9	34.7	1.3	33.2–36.4
Tibia	19.8	1.8	17.2–21.3	18.1	1.0	17.2–19.5
Foot	16.0	0.8	14.9–16.9	14.8	0.9	13.4–15.9
Head width	11.8	0.4	11.2–12.2	11.7	0.5	11.2–12.2
Head length	12.6	0.9	11.3–13.5	12.4	0.8	11.7–13.7
Eye diameter	3.3	0.2	3.1–3.6	3.4	0.5	2.8–4.0
Tympanum diameter	2.4	0.2	2.1–2.7	1.9	0.2	1.7–2.3

All measurements are in mm.

#### Comparison with other species

*Scinax quinquefasciatus* is most similar to *Scinax tsachila* sp. nov. but differs by the presence of scattered to abundant small tubercles on the dorsum (tubercles are absent in *S*. *tsachila*). *Scinax tsachila* sp. nov. is also distinct in having green shank bones visible through the skin (white to bluish-white bones in *S*. *quinquefasciatus*). *Scinax sugillatus* is sympatric with *S*. *quinquefasciatus* in western Ecuador. It is readily distinguished by its larger size (average SVL 39.9 mm in males and 45.5 mm in females; [52]), by the presence of a row of tubercles on the lower jaw (absent in *S*. *quinquefasciatus*), and by having distinctive black and blue mottling in the groin and on the anterior and posterior surfaces of the thighs (mottling absent in *S*. *quinquefasciatus*). *Scinax ruber* is an abundant species in Amazonian Ecuador. It differs from *S*. *quinquefasciatus* in having posterior surfaces of the thighs black with yellow spots (brown with paler marks in *S*. *quinquefasciatus*).

#### Redescription of the holotype

**Fig 3:**
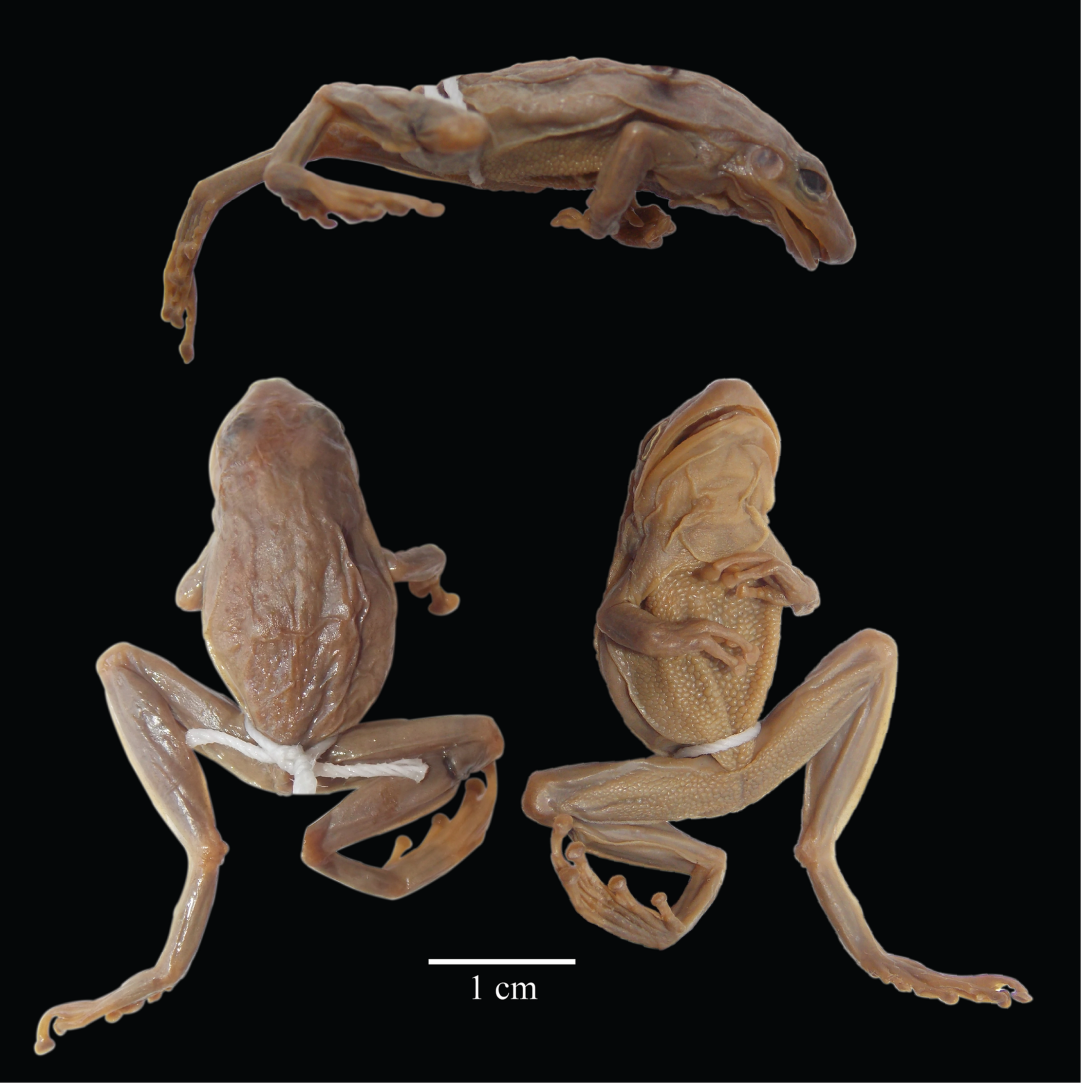
Holotype of *Scinax quinquefasciatus*. Lateral, dorsal, and ventral views. ANSP 18115, 30.0 mm SVL. Photographs by Ned Gilmore.

An adult male, 30.0 mm SVL; body moderately robust (Fig 3); snout acutely rounded in dorsal view and in profile; eye-nostril distance slightly less than diameter of eye; nostrils not protuberant slightly behind anterior margin of lower jaw; internarial region barely depressed; canthus rostralis rounded; loreal region barely concave; lips thin, rounded; top of head flat; interorbital distance much greater than width of eyelid; supratympanic fold moderately robust obscuring upper edge of tympanic annulus; tympanum round, its diameter 74% of diameter of eye, tympanic annulus distinct. Forelimb slender; ulnar tubercles absent; palmar tubercle bifid; prepollical tubercle round; subarticular tubercles large, round; supernumerary tubercles large, in two rows proximally; fingers moderately long bearing slightly truncated terminal discs; relative lengths of fingers from shortest to longest I, II, IV, III; outer fingers webbed basally; webbing formula **II**2—3**III**3—2**IV**; nuptial excrescences absent. Hind limb moderately slender; tibia length 52% SVL; foot length 43% SVL; tubercles and calcar on heel absent; inner tarsal fold weak on distal two-thirds of tarsus; inner metatarsal tubercle elliptical, not visible from above; outer metatarsal tubercle small, conical; toes bearing rounded terminal discs slightly smaller than those on fingers; relative lengths of toes from shortest to longest I, II, V, III, IV; outer toe about two-thirds webbed; webbing formula **I**11/2–21/2**II**11/2—2**III**11/2—2**IV**2—1**V**; subarticular tubercles large, conical; supernumerary tubercles large, present on proximal digits. Skin on dorsum smooth; skin on venter granular; thoracic fold absent; vocal sac single, median, subgular; cloacal sheath short; cloacal folds and tubercles absent; tongue cordiform; internal choanae large, ovoid; 6–5 vomerine teeth in transverse row between choanae; vocal slits extending from mid-lateral base of tongue to angle of jaw.

*Measurements of holotype (in mm)*. SVL 30.0, tibia length 15.5, foot length 13.0, head width 9.5, head length 10.8, interorbital distance 3.2, width of eyelid 2.5, eye–nostril distance 3.2, internarial distance 2.0, diameter of eye 3.5, diameter of tympanum 2.6.

*Color of holotype in preservative*. Fowler ([53]:161) described the “Color in alcohol largely dull or pale brownish above, lighter or paler below, and of uniform tint. Back with five lengthwise darker streaks, median vertebral as triangle between eyes, and extends on the front of the upper eyelids. From posterior surfaces of latter each outer streak extends back, while outermost includes tympanum and runs well lateral along body. Upper surfaces of limbs with well-defined cross-ands, especially on femora and tibia. Hind surfaces of femora mottled slightly with dusky.” Fowler’s description agrees with his figure of the holotype (:Pl. VII). When Duellman examined the holotype on 11 June 1969 the dorsum was tan with darker tan markings; only two fragmented, longitudinal stripes were obvious on the dorsum of the body. No markings were visible on the head or on the dorsal surfaces of the limbs. The ventral surfaces were uniform tan, slightly paler than the dorsum. Faint intrusions of the dorsal color were evident on the posterior surfaces of the thighs.

#### Variation

Descriptive statistics of morphometric measurements are given in Table 3. There is variation in the nature of the inner tarsal fold and in the texture of the skin on the dorsal surface of the body. In 37 specimens (84.1%) a tarsal fold is not evident. In three individuals the fold exists on the distal fourth of the tarsus, and in three others it extends to the mid-length of the tarsus, whereas the fold is barely evident on the distal two-thirds of the tarsus (same extent as in the holotype). A brown canthal stripe and cream or white labial stripe are present in all individuals. A brown triangular mark is present on the head in all specimens. The triangle is on the occipital region of the head; the base extends to the outer edges of the eyelids, and the apex is directed posteriorly on, or about, the mid-line. In some specimens (e.g., QCAZ 27019) a smaller triangle is present anterior to the level of the orbits. Twenty specimens (45.6%) have five longitudinal brown stripes on the body. The median stripe usually is connected to the triangular mark on the head; the paravertebral stripes originate posterior to the eyelids, and the lateral stripes are continuous with postorbital stripes on the head. Nineteen specimens (43.2%) have only three stripes; the lateral stripes are absent. Five individuals (11.4%) have only paravertebral stripes. Transverse brown marks are present on the dorsal surfaces of the hindlimbs in most specimens. These marks are distinct in 15 specimens (34.1%), whereas they are weakly defined in 24 specimens (54.5%). Markings on the hind limbs are absent in five specimens (11.4%).

Three recently metamorphosed juveniles with clusters of melanophores on either side of the terminus of the urostyle have SVLs of 13.5–15.2 mm ($\bar{x}$ = 14.5 mm). Four subadults have SVLs of 20.0–30.4 mm.

#### Color in life

**Fig 4:**
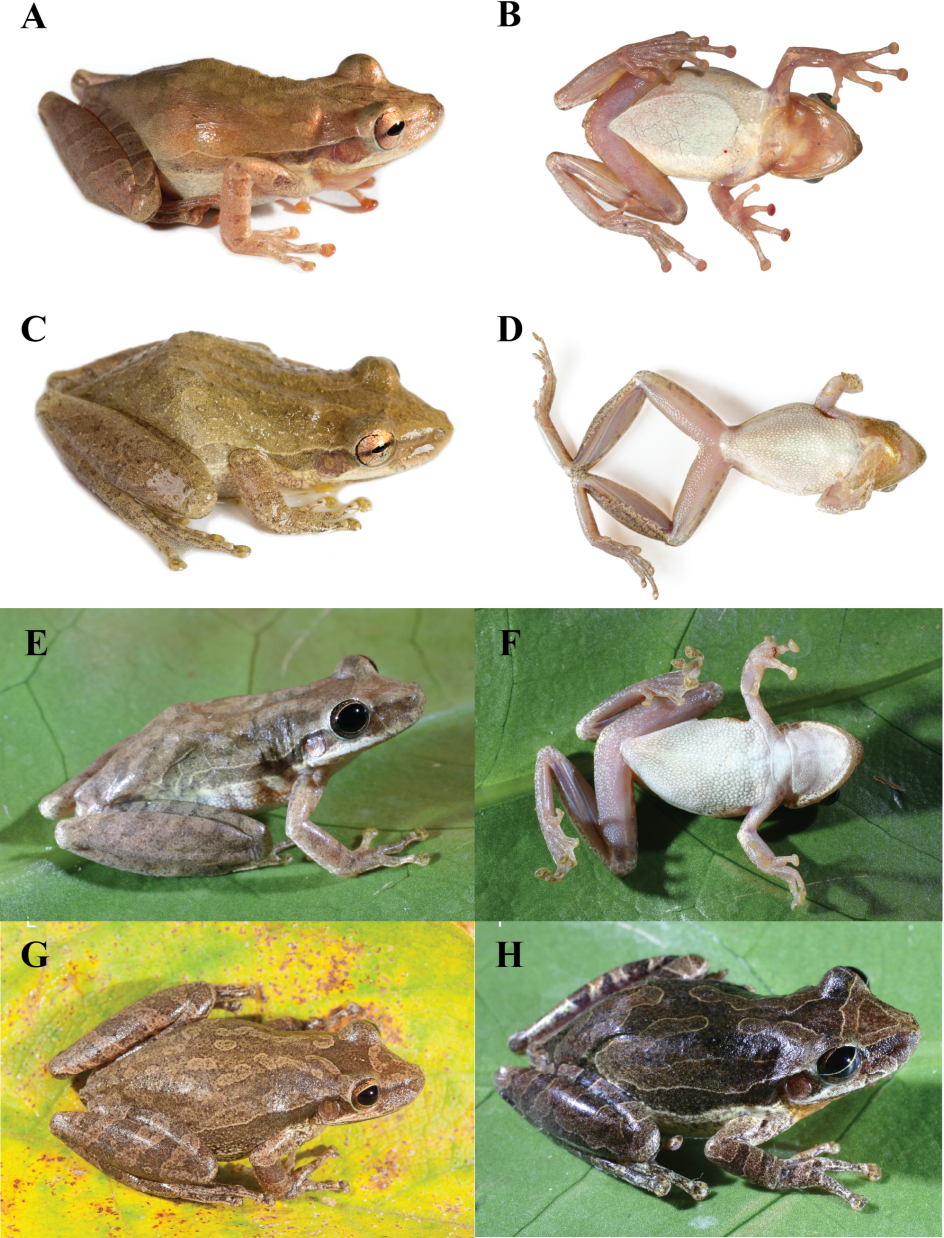
*Scinax quinquefasciatus* in life. A–B. QCAZ 50681, adult male, SVL 34.4 mm, road between Rio Coaque and Jama, Provincia Manabí, Ecuador. C–D. QCAZ 55637, adult male, SVL 29.6 mm, Huaquillas, Provincia El Oro, Ecuador. E–F. QCAZ 27018 adult female, SVL 34.2 mm, Puyango River in the Alamor-Arenillas road, Provincia El Oro, Ecuador. G. QCAZ 42255, adult male, SVL 38.7 mm, Centro Científico Río Palenque, Provincia Los Ríos, Ecuador. H. QCAZ 27019, adult male, SVL 33.6 mm, Puyango River in the Alamor-Arenillas road, Provincia El Oro, Ecuador. All photographs by S. R. Ron.

Variation is shown in Fig 4. Dorsal coloration varies between dark brown and pale brown with darker marks arranged in irregular patterns. Dark marks almost always include a brown triangular mark on the head but one non-collected individual photographed at Puerto Villamil, Provincia Galapagos, lacks that mark. Posterior surfaces of the thighs vary between brown and pale brown with pale brown to yellowish cream marks (Fig 4). There is a dark brown lateral longitudinal band starting at the tympanum and extending to the mid-flank (Fig 4E) or even to the groin (Fig 4H).

#### Advertisement call

**Fig 5:**
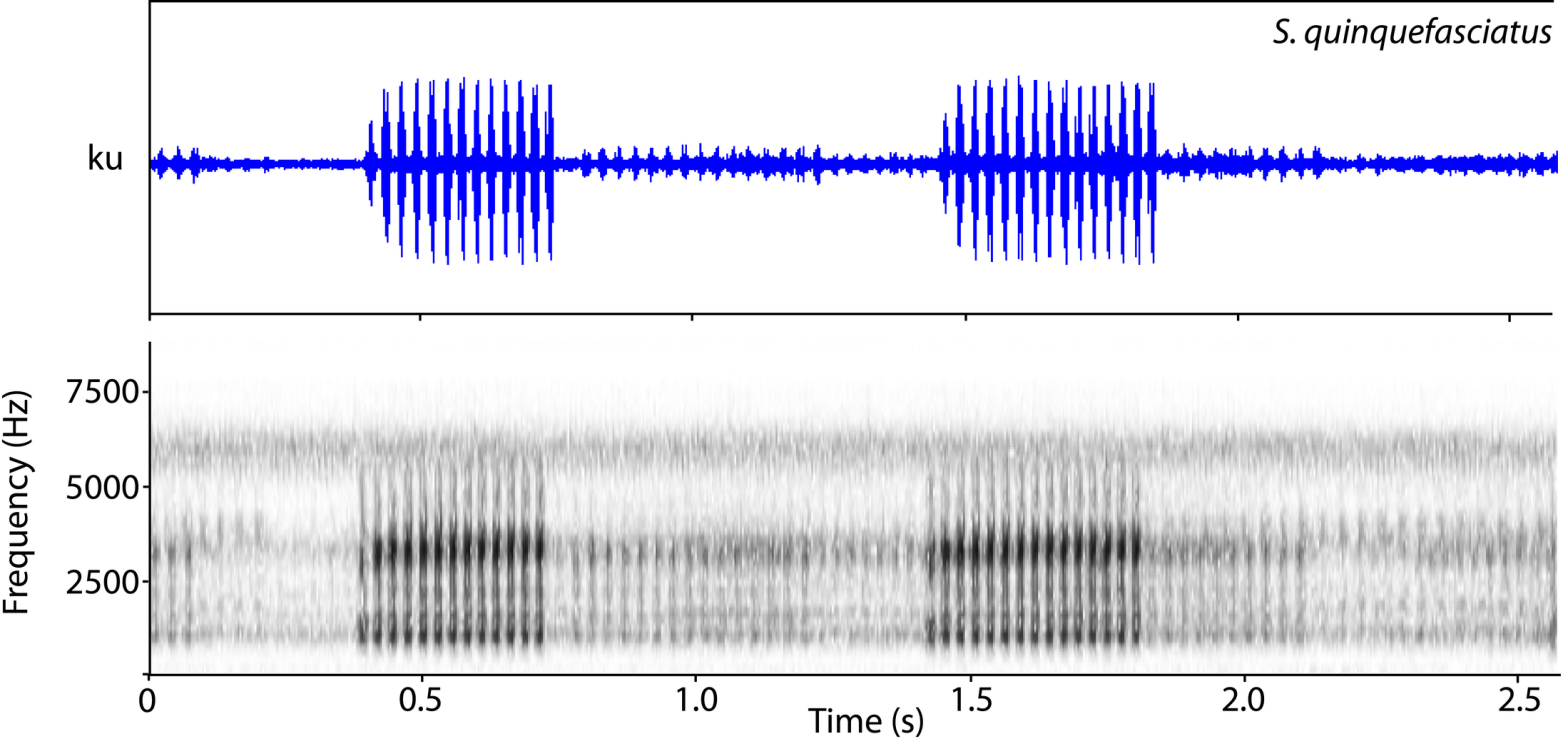
Advertisement call of *S*. *quinquefasciatus*. Sound spectrogram (below) and corresponding oscillogram (above). Call voucher QCAZ 50704 from 5 km N from Rocafuerte, Provincia Manabí, Ecuador.

This description is based in calls from two males, QCAZ 23378 and QCAZ 50704 (Table 4). The call (Fig 5) is loud and pulsed with duration varying from one third of a second to one second. Most call energy is distributed among four frequency bands of which either the second or the third have the dominant frequency.

**Table 4:** Descriptive statistics and results from Students *t*-tests for calls of *Scinax*.

	*S*. *quinquefasciatus* (n = 3)	*S*. *elaeochroa* (n = 7)	*S*. *tsachila* sp. nov. (n = 5)	*S*. *elaeochroa* vs. *S*. *tsachila* sp. nov.
Call duration (s)	0.52 ± 0.09 (0.43–0.61)	0.17 ± 0.07 (0.08–0.30)	0.15 ± 0.02 (0.13–0.17)	*t* = -0.83, df = 7 P = 0.42
Rise time (s)	0.28 ± 0.12 (0.15–0.38)	0.13 ± 0.07 (0.06–0.26)	0.09 ± 0.03 (0.04–0.12)	*t* = -1.48, df = 8.6 P = 0.17
Fundamental frequency (Hz)	1055.76 ± 87.98 (955.4–1119.7)	1487.55 ± 61.22 (1378.1–1576.3)	1452.19 ± 54.76 (1359.4–1504.7)	*t* = -1.04, df = 9.3 P = 0.32
Dominant frequency (Hz)	3287.6 ± 484.5 (2728.3–3574.5)	2184.1 ± 891.9 (1378.1–3186.9)	1452.19 ± 54.8 (1359.4–1504.7)	*t* = -2.16, df = 6 P = 0.07
Pulse number	17.80 ± 2.80 (11–31)	6.86 ± 2.31 (3–11)	9.21 ± 1.10 (8–11)	***t* = 2.34, df = 9** **P = 0.04**
Pulse rate (pulses/call duration)	37.64 ± 4.63 (34.46–42.95)	40.73 ± 4.64 (32.41–46.16)	62.05 ± 2.48 (59.54–65.76)	***t* = 10.26, df = 9.4** **P < 0.001**
Call rate (calls per second)	0.59 ± 0.26 (0.32–0.84)	1.20 ± 0.36 (0.76–1.76)	0.95 ± 0.28 (0.70–1.31)	*t* = -1.37, df = 9.8 P = 0.19

Voucher specimens: *S*. *quinquefasciatus* QCAZ 23378, 50704; *S*. *elaeochroa* KU 64422–23, 64478–79, 64501, 93940–41, and *S*. *tsachila* sp. nov. QCAZ 23672–73. The *n* values indicate the number of males analyzed. Mean ± SD is given with range in parentheses.

#### Distribution and ecology

*Scinax quinquefasciatus* occurs in the Pacific Basin of Ecuador and southwestern Colombia [54]. Specimens with elevation data range from 0 to 620 m above sea level (upper limit near Pedro Vicente Maldonado, Provincia Pichincha, Ecuador). It occurs in Chocoan Tropical Rainforest, Andean Western Foothill Forest, Deciduous Forest, and Dry Costal Shrub (natural regions as defined by [55]). It thrives in artificial open areas including agricultural fields, swamps, small lakes, pastures, and suburban areas. Males call while perched on vegetation next to ponds, small lakes, swamps, or flooded rice fields (QCAZ database, available at https://bioweb.bio/portal/).

*Scinax quinquefasciatus* is an invasive species in the Galapagos Archipelago in towns and farms in Isabela, Santa Cruz, and San Cristobal Islands [56]. However, no records have been reported for San Cristobal since 2001. The first specimen was collected in 1973 in Santa Cruz Island, but its confirmed establishment, associated with increased humidity and rainfall due to a strong El Niño event, occurred later in 1998 in Isabela Island at Poza Las Diablas, near Puerto Villamil [57]. Despite the limited evidence of its effect on Galapagos biodiversity, Phillips et al (2012) suggested predation of native invertebrate fauna as a potential impact, given the insectivorous nature of the species. Eradication programs carried out by the Galapagos National Park Service, including hand-capture, caffeine spraying, and change of the lagoons’ salinity, have been unsuccessful [57,58].

#### Conservation status

**Fig 6:**
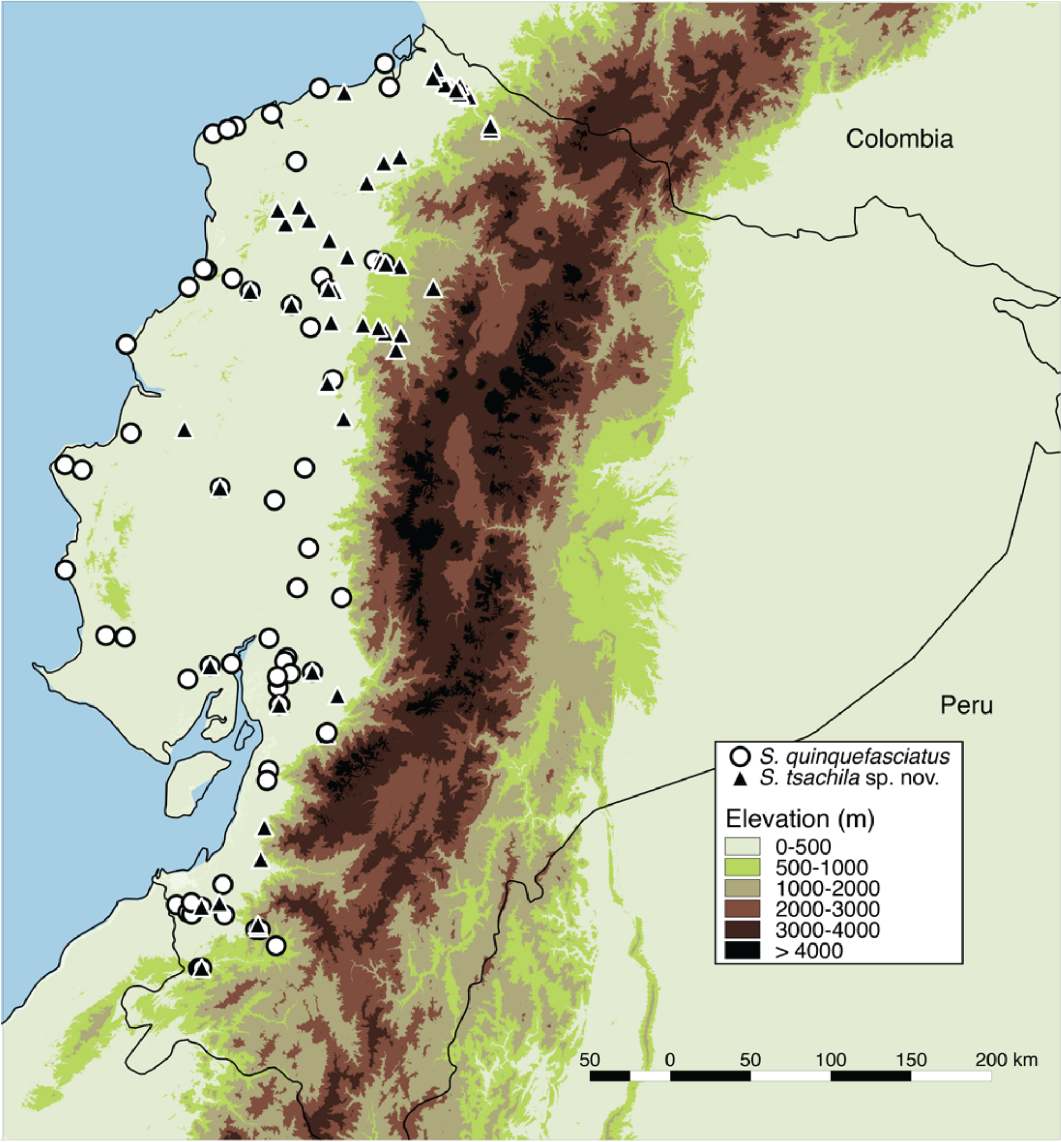
Distribution map of *Scinax quinquefasciatus* and *S*. *tsachila* sp. nov. Locality data from specimens deposited at Museo de Zoología of Pontificia Universidad Católica del Ecuador (QCAZ), Quito.

*Scinax quinquefasciatus* is an abundant species in artificial open areas, the habitat type that covers most of the Pacific Basin of Ecuador, below 600 m [59]. It can be locally abundant and is widely distributed (Fig 6). Given its wide distribution, tolerance of anthropogenic habitat disturbance, and local abundance, we recommend maintaining it in the Least Concern category (based on Red List criteria, [60]. Its colonization and spread to the Galapagos islands demonstrate its potential as an invasive species. Control programs should be implemented to prevent its movement in ships and its establishment outside their native range. Inadvertent movement of this species by humans is likely facilitated by its frequent presence in human dwellings throughout its native range.

#### Remarks

As noted by Duellman ([51]: 217), Fowler’s designation of the type locality is erroneous. The holotype was obtained by Samuel N. Rhoads, whose field label in the jar with the holotype reads “*Hyla* Duran, Ec., 2/12/1911.”

### *Scinax tsachila* sp. nov.

urn:lsid:zoobank.org:act:95590544-ECF7-469F-8376-B129BE5799DA

#### Holotype

QCAZ 39880, an adult male, collected by Samael Padilla, 4 km S from Santa Rosa, on the road to Las Balsas, 44 m above sea level, Provincia El Oro, Ecuador; 13 February 2009 at 21:00 h.

#### Paratypes

All from Ecuador: Provincia El Oro: 1 km from Río Puyango bridge on Alamor-Arenillas road, QCAZ 27017; road between Huaquillas and La Cuca Arenillas, QCAZ 23672–73, 23678. Provincia Esmeraldas: Durango, QCAZ 26102, 26104, 45423–24; Playón San Franciso, QCAZ 40843. Provincia Los Ríos: Centro Científico Río Palenque, 56 km N Quevedo, KU207379–80; 6.9 km N Babahoyo, KU 21840–82. Provincia Manabí: 4.6 km W El Carmen, KU 218490; 20 km W El Carmen, QCAZ 23183; 52 km W El Carmen, QCAZ 23184–85. Provincia Pichincha: Los Bancos, KU 212672l; Puerto Quito, KU 212571; 1 km E Vincente Maldonado, KU 218491–502. Provincia Santo Domingo de los Tsáchilas: La Concordia, Bosque Protector La Perla, KU 218506; 1 km W La Concordia, KU 218503–05; Nueva Israel, QCAZ 23175.

#### Diagnosis

(1) Average SVL in males 31.6 mm (range 27.2–34.2), in females 34.7 mm (range 33.2–36.4), Table 3; (2) snout acutely rounded in dorsal view and in profile; (3) ulnar and tarsal tubercles absent; (4) enlarged heel tubercle absent; (5) tubercles absent on lower jaw; (6) skin on dorsum smooth to shagreen without scattered tubercles; (7) diameter of tympanum 15.0% of head length; (8) dorsum cream to reddish brown with or without longitudinal brown stripes; (9) flanks lacking patterns; (10) posterior surfaces of thighs without markings; (11) iris brown with orange flecks to orange-yellow with brown reticulations.

#### Comparison with other species

**Fig 7:**
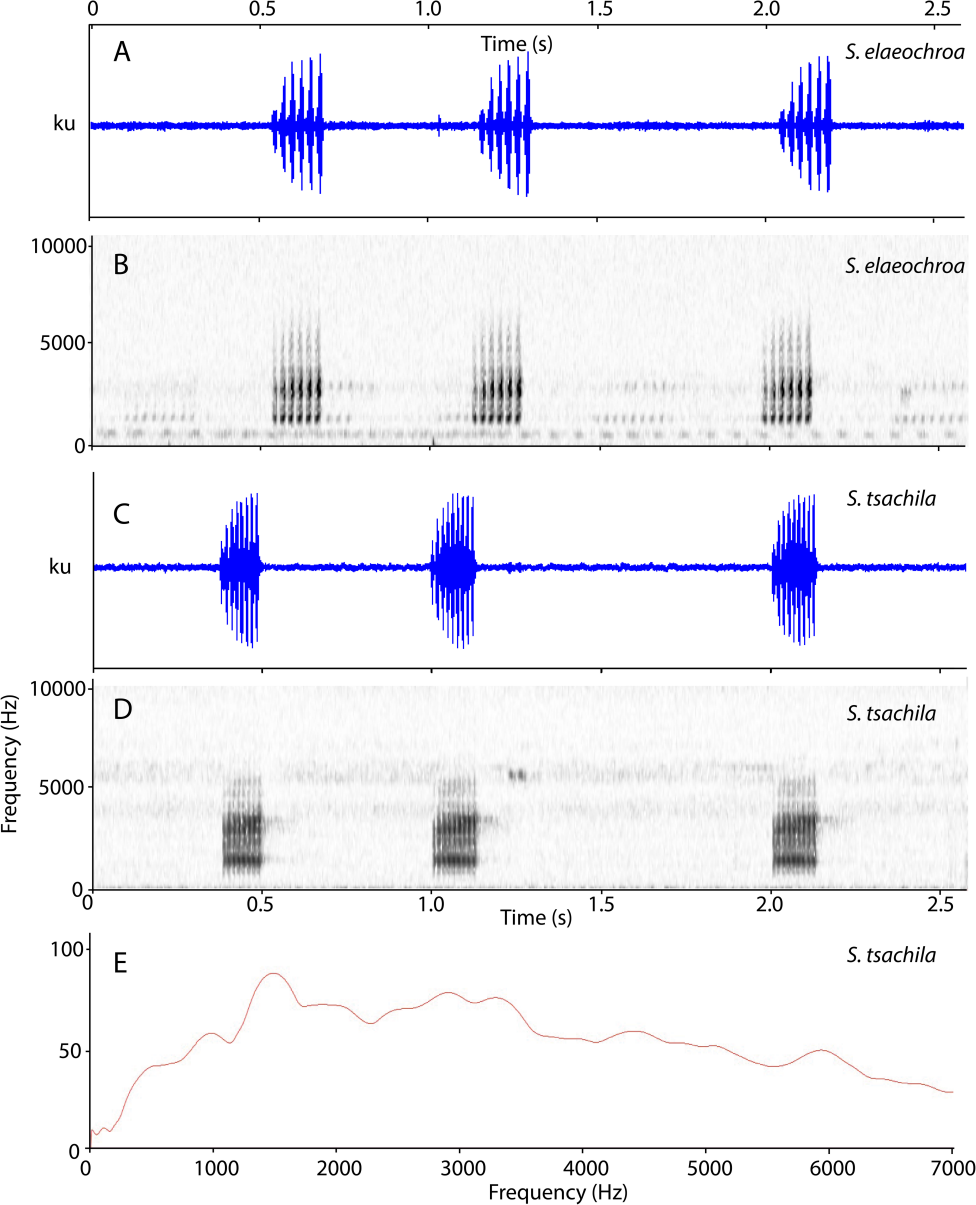
Advertisement calls of *Scinax elaeochroa* and *Scinax tsachila*. A and C are oscillograms, B and D are spectrograms, E is a power spectrum. Call vouchers: *S*. *elaeochroa* KU 64422 from Turrialba, Provincia Cartago, Costa Rica; *S*. *tsachila* QCAZ 23673 from Arenillas, in the Huaquillas-La Cuca road, Provincia El Oro, Ecuador.

**Fig 8:**
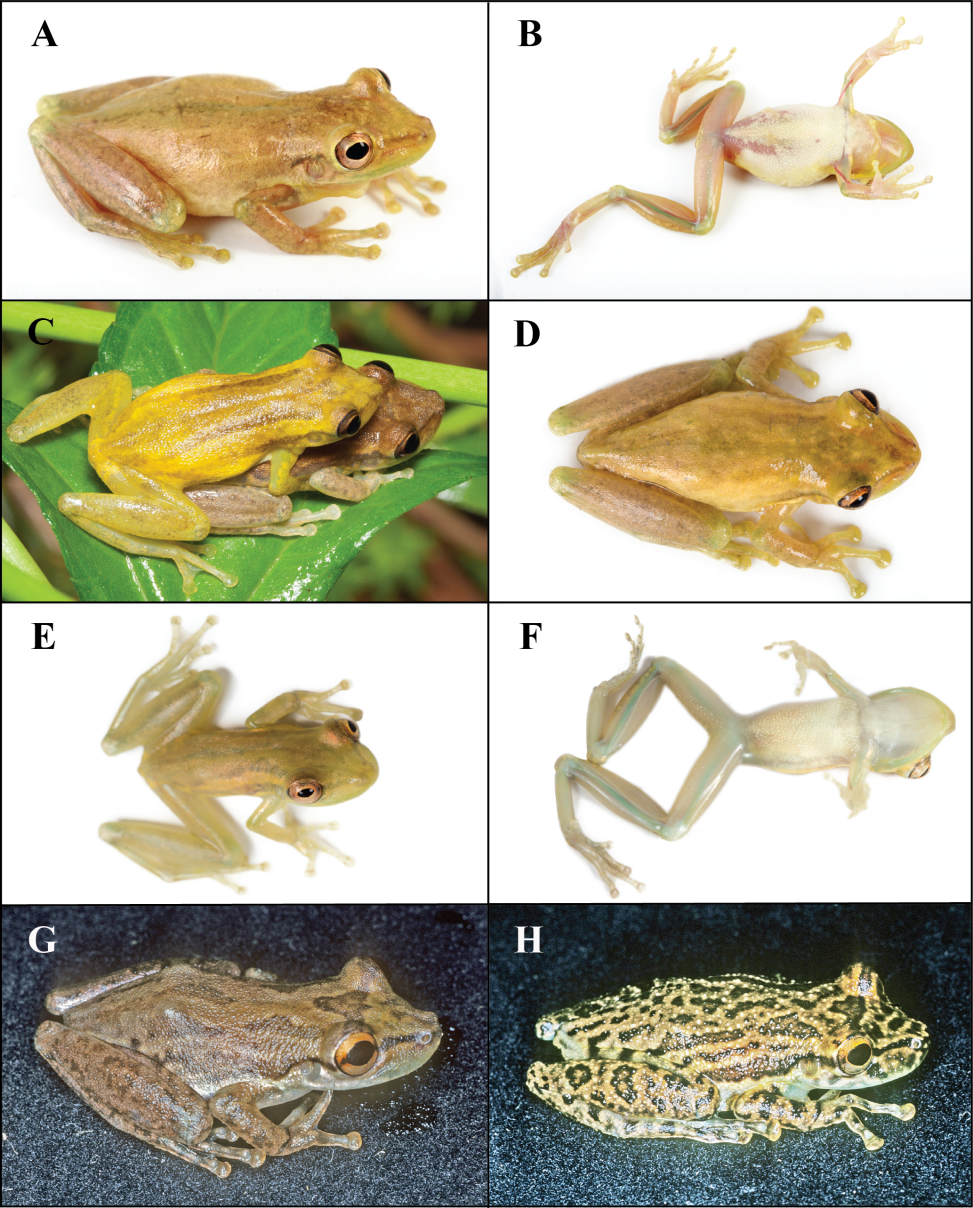
*Scinax tsachila* and *S*. *elaeochroa* in life. A–F *Scinax tsachila*, G–H *S*. *elaeochroa*. A–B. QCAZ 39880, holotype, adult male, SVL 27.2 mm, Santa Rosa, Provincia El Oro, Ecuador. C. Amplectant pair, QCAZ 40848, adult male SVL 31.2 mm; QCAZ 40849, adult female, SVL 32.1 mm, Playón de San Francisco, Provincia Esmeraldas, Ecuador. D. QCAZ 62975, adult male, SVL 32.7 mm, vía Zaracay-Piñas, Provincia El Oro, Ecuador. E–F. QCAZ 57045, subadult female, SVL 24.6 mm, Tundaloma Lodge, Provincia Esmeraldas, Ecuador. G. KU 64336, adult male, 35.3 mm SVL, Quebrada Boruca, Puntarenas, Costa Rica. H. KU 64499, adult male, 30.7 mm, Laguna Monte Alegre, Alajuela, Costa Rica. Photos A–F by S. R. Ron, GH by W. E. Duellman.

*Scinax tsachila* is most similar to *S*. *quinquefasciatus*, *S*. *elaeochroa* and the recently described *S*. *caprarius*. *Scinax tsachila* differs from *S*. *quinquefasciatus* in having a different advisement call (Figs 5 and 7) and distinct skin texture in the dorsum (in life): scattered to abundant small tubercles in *S*. *quinquefasciatus* vs. tubercles absent in *S*. *tsachila*. Both species also differ in bone coloration: in the ventral face of the shank, in *S*. *quinquefasciatus*, bones are white and barely visible externally through the skin; in *S*. *tsachila*, bones are green and evident against the background (Fig 8F). *Scinax tsachila* differs from *S*. *elaeochroa* in advertisement call (Fig 7); pulse number and pulse rate are significantly different between both species (Table 4). In addition, both species have distinct coloration with most *S*. *elaeochroa* having a dark interorbital triangular mark and bars on the dorsal surfaces of the limbs [20,61] which are absent or are faint in *S*. *tsachila*. In 104 specimens of *S*. *tsachila*, 13.4% have dark marks on the dorsal surfaces of the limbs and ~10% have a dark interorbital triangular mark which is less conspicuous than in *S*. *elaeochroa* (Fig 8A vs. 8G-H). *Scinax tsachila* differs from *S*. *caprarius* in having smooth dorsal skin (tuberculate in *S*. *caprarius* [62]) and an advertisement call with lower dominant frequency (1359–1505 Hz in *S*. *tsachila* vs. 2184–3218 in *S*. *caprarius* [62]).

*Scinax sugillatus* also occurs in western Ecuador. It is readily distinguished by its larger size (average SVL 39.9 mm in males and 45.5 mm in females; [52]), by the presence of a row of tubercles on the lower jaw (absent in *S*. *tsachila*), and by having distinctive black and blue mottling in the groin and on the anterior and posterior surfaces of the thighs (mottling absent in *S*. *tsachila*).

Four members of the genus occur in Amazonian Ecuador. Of these, *Scinax ruber* differs by having posterior surfaces of the thighs black with yellow spots (reddish brown, without pattern in *S*. *tsachila*). *Scinax cruentomma* is distinct in having a horizontal red bar in the iris (absent in *S*. *tsachila*). *Scinax funereus* can be recognized by its tuberculate dorsum (shagreen in *S*. *tsachila*). *Scinax garbei* has a row of tubercles on the lower jaw, a large tubercle on the heel, and black and yellow bars on the posterior surfaces of the thighs (all absent in *S*. *tsachila*).

Comparative data for the following section is based on Ron et al. [55]. Of the nine other species of hylid frogs on the Pacific lowlands in Ecuador, all are much larger than *Scinax*, except *Dendropsophus gryllatus*, which has an axillary membrane and a yellow dorsum with a large mid-dorsal brown mark. *Boana pellucens* and *B*. *rubracyla* are much larger (*B*. *pellucens* SVL to 52.8 mm in males and 60.3 mm in females; *B*. *rubracyla* SVL to 50.5 in males and 59.0 in females) and are green dorsally; males have a projecting prepollical spine. The latter feature also is characteristic of two other large species: *Boana boans* (SVL in males to 118 mm, females to 110) and *B*. *rosenbergi* (males to 90.9 mm, females to 97.8). These are predominantly brown frogs with nearly fully webbed fingers and toes. *Boana picturata* is larger (SVL in males to 52.7 mm, females to 69.4 mm) and have proportionally larger eyes. Webbing extends at least half the lengths of the fingers in four large (SVLs in males more than 60 m) species. Of these, *Agalychnis spurrelli* is green and has a vertical pupil, and *Smilisca phaeota* is green or tan with a large brown mid-dorsal blotch. In males of the other two species—*Trachycephalus jordani* and *T*. *quadrangulum* (formerly *Phrynohyas venulosa*)—the vocal sacs are paired and located behind the angles of the jaws. The former species is nearly uniform brown dorsally and has a casqued head with the skin co-ossified with the underlying dermal bones. *Trachycephalus quadrangulum* has thick, glandular skin on the dorsum, which is brown with conspicuous dark marks.

#### Description of holotype

Body slender, slightly wider than head; snout acutely rounded in dorsal view and in profile; eye-nostril distance slightly less than diameter of eye; nostrils barely protuberant at level of anterior margin of lower jaw; internarial region barely depressed; canthus rostralis rounded; loreal region barely concave; lips rounded; top of head flat; interorbital distance much greater than width of eyelid; supratympanic fold weak, barely obscuring upper edge of tympanic annulus; tympanum round. Forelimb moderately short; ulnar tubercles absent; fingers moderately long bearing transversely rounded terminal discs; palmar tubercle small, diffuse; thenar tubercle elliptical; subarticular tubercles prominent, subconical, nearly as wide as digit; supernumerary tubercles rounded, numerous; relative lengths of fingers I < II < IV < III; webbing absent between Fingers I and II, basal between Fingers II and III, absent between Fingers III and IV; narrow lateral keels on Fingers II, III, and IV; nuptial excrescence not evident. Hind limb sender; tibia length 47.8% of SVL; tubercles and calcar absent on heel; foot length 42.6% of SVL; inner tarsal fold absent; inner metatarsal tubercle ovoid, visible from above; outer metatarsal tubercle small, rounded; relative lengths of toes I < II < III < IV < V; subarticular tubercles small, rounded; supernumerary tubercles small, present on proximal segments of Toes II–V; webbing basal between Toes I and II; other toes about two-thirds webbed; webbing formula **II**1—2**III**1—2**IV**2—1**V**; terminal discs on toes rounded, slightly smaller than those of fingers. Skin on all dorsal surfaces and flanks weakly shagreen; skin on belly and median ventral surfaces of thighs granular; skin on other ventral surfaces smooth; cloacal sheath short; cloacal opening directed posterior at upper level of thighs; minute subcloacal tubercles present. Vocal sac single, median, subgular; vocal slit extending from midlateral base of tongue nearly to angle of jaw; tongue narrowly cordiform, shallowly notched posteriorly, free posteriorly for no more than one-fifth of its length; dentigerous processes of vomers transverse between ovoid choanae, each bearing five teeth.

*Measurements of holotype (in mm)*. SVL 27.2, tibia length 13.0, foot length 11.6, head width 8.3, head length 9.8, interorbital distance 3.8, width of eyelid 2.3, eye–nostril distance 3.0, internarial distance 1.8, diameter of eye 3.4, diameter of tympanum 1.4.

**Fig 9:**
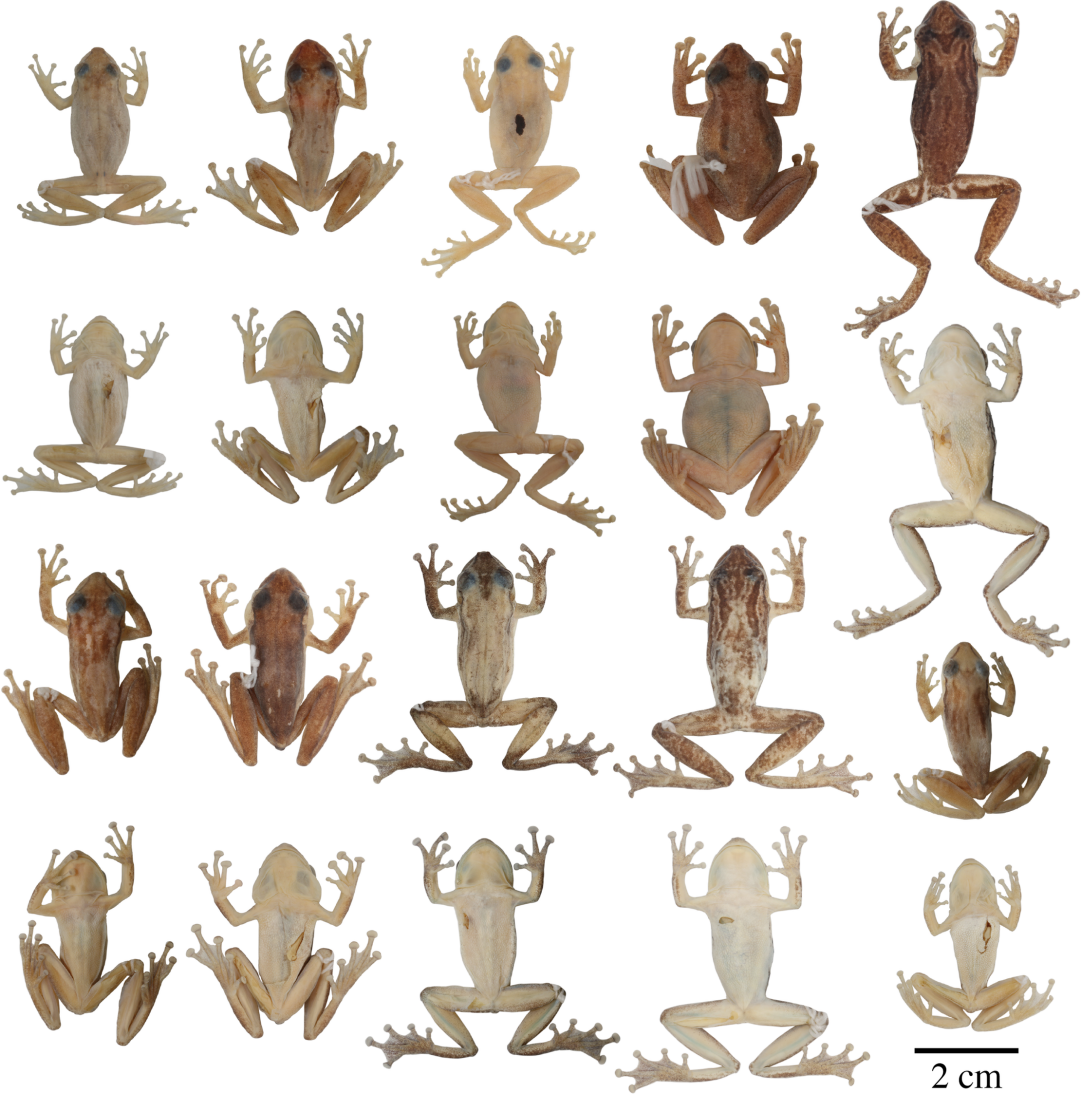
Adult preserved specimens of *Scinax tsachila* showing variation in dorsal and ventral coloration. From left to right, first and second rows: QCAZ 39880 (holotype), 23184, 27629 (males), 30764 (female), 62535 (male); third and fourth rows: QCAZ 40843 (female), 23175, 66642 (males), 65690 (female), 23673 (male). See listed in the Appendix I for locality data. All specimens are shown at the same scale. Photographs by Gustavo Pazmiño and M. Caminer.

*Color of holotype in preservative*. (Fig 9) Dorsum creamy tan with a faint brown, narrow middorsal stripe extending from occiput to sacrum; two faint brown stripes extending posteriorly from inner edge of eyelid, becoming diffuse at about mid-length of body; no markings on limbs or flanks. Dark brown canthal stripe present, ventral surfaces white except for faintly yellow vocal sac.

*Color of holotype in life*. (Fig 8A and 8B) Dorsal surfaces of head, body, and limbs pale reddish brown with faint darker brown canthal and supratympanic stripes; faint mid-dorsal longitudinal brown stripe barely evident; extremely faint irregular, transverse, dark brown marks on dorsal surfaces of hindlimbs; belly white; other ventral surfaces unpigmented; bones green; iris brown with orange flecks.

#### Variation

Morphometric variation is shown in Table 3. Adult males are smaller than females (male SVL/female SVL = 0.84). Texture of the skin on the dorsal surface of the body varies from smooth (in about two-thirds of the individuals) to shagreen (one third, 35.4%; n = 48). Variation in color pattern of preserved specimens is shown in Fig 9. Background dorsal coloration varies from creamy tan (e.g., QCAZ 27629) to pale grayish brown (e.g., QCAZ 66642), brown (e.g., QCAZ 30764), or reddish brown (e.g., QCAZ 40843). In some individuals there are no visible dark markings on the body (e.g., QCAZ 23175, 66642). In other specimens the pattern consists of three stripes (mid-dorsal and two paravertebral) (e.g., QCAZ 66647), whereas the mid-dorsal stripe can be absent in some specimens that have only paravertebral stripes (e.g., QCAZ 30764). The longitudinal brown stripes usually originate in the occipital region and extend toward the sacral region (e.g., QCAZ 62537). Some specimens have five stripes (e.g., QCAZ 26102); the lateral ones are extensions of a postorbital stripe. All specimens have a narrow brown canthal stripe with sometimes an interorbital stripe or mark (e.g., QCAZ 23673, 30764). A dark interorbital triangular mark (e.g., QCAZ 62535) is present in ~10% of the individuals. A pale labial stripe usually extends from below the eye to the posterior end of the jaw. Dark brown transversal or irregular marks can be present on the thighs and shanks (e.g., QCAZ 62535, 65690), whereas these marks are faint or absent in other specimens (e.g., QCAZ 23184, 27629). The posterior surfaces of the thighs are uniform pale cream to white in all specimens.

#### Advertisement call

Based on recordings of QCAZ 23672–73 (26 March 2003; 21h55, 25.4 ºC) and three uncollected males (31 March 1967 and 12 April 1972; 20h50; 20.5–25 ºC). The advertisement call consists of single short-pulsed notes with an average duration of 0.15 s (range 0.13‒0.17 s) and 8–11 pulses per note (Fig 7). The dominant frequency is equal to the fundamental and has an average of 1452.19 Hz (range 1359.40‒1504.68 Hz). The mean rise time is 0.09 s (range 0.04‒0.12 s) and the call rate is 0.95 s (range 0.70‒1.31 s) (Table 4).

#### Distribution and ecology

*Scinax tsachila* occurs in the Pacific Basin of Ecuador. Specimens with elevation data range from 0 to 1207 m above sea level (near Mindo, Provincia Pichincha; Fig 6). The available evidence suggests the occurrence of *S*. *tsachila* in Colombia. Records of “*S*. *elaeochroa*” from, Nariño Department, Colombia, [62,63] likely represent *S*. *tsachila* as some of they are only 70 km from *S*. *tsachila* populations in Ecuador (see Discussion).

**Fig 10:**
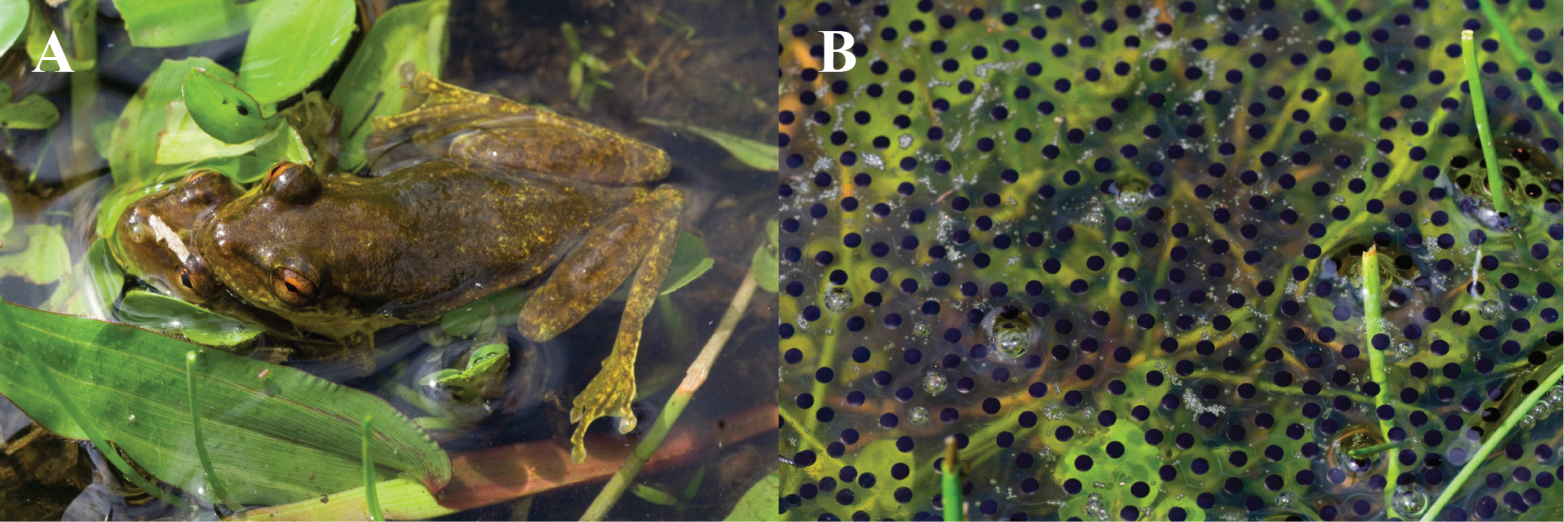
Reproduction in *Scinax tsachila*. A. Amplectant pair (not collected). B. Eggs. Photos by Pol Pintanel. Ecuador, Provincia Pichincha, Mindo, near the juncture of Río Cinto and Río Seloya.

*Scinax tsachila* occurs in the following natural regions: Chocoan Tropical Rainforest, Andean Western Foothill Forest Deciduous Forest, and Dry Costal Shrub (natural regions as defined by [55]). It is found most frequently in artificial open areas including agricultural fields, pastures, house backyards, and even buildings. Few individuals were found in secondary forest. Out of 167 georeferenced specimens at the QCAZ collection, 144 (86%) were found in artificial open areas (“*intervención*” category on [64] map); the remaining fell in the “forest” category. Males call from the ground or while perched on low vegetation on puddles, ponds, small lakes, or swamps in open areas (QCAZ database). Two males (QCAZ 39880, 42289) were calling from puddles formed on cow footprints. At 20 km NW from El Carmen, Provincia Manabí, *S*. *tsachila* and *S*. *quinquefasciatus* were part of the same chorus (SRR field notes). Amplexus is axillary and eggs are deposited on water (Fig 10). One adult male (QCAZ 23619) was regurgitated by a snake (*Leptodeira*).

#### Conservation status

*Scinax tsachila* can be abundant in artificial open areas, the habitat type that covers most of the Pacific Basin of Ecuador [59]. Increasing deforestation in Ecuador should benefit populations of this species. Therefore, we suggest assigning *S*. *tsachila* to the Least Concern category.

#### Etymology

The specific name is a noun in apposition. The epithet refers to the Tsáchila people who inhabit the area within the range of *Scinax tsachila* in Ecuador. Men in this ethnic group mold their hair into a helmet-like shape that is dyed red with the juice of the achiote bush (Bixaceae: *Bixa orellana*). The Spaniards called them “Colorados” (colored red). Hence the former name of the major population center in the area, Santo Domingo de los Colorados.

#### Remarks

A potential senior synonym for *S*. *tsachila* is *Hyla dulcensis* [65]. Its type locality is “Golfito, Puntarenas Province, Costa Rica”. *Hyla dulcensis* was considered a junior synonym of *S*. *elaeochroa* by Duellman [66]. Examination of the holotype of *H*. *dulcensis* (KU 32168) confirms that it is not conspecific with *S*. *tsachila*. The holotype has the characteristic dark interorbital triangular mark present in most *S*. *elaeochroa*. This is supported by the phylogeny which shows that samples of *S*. *elaeochroa* from Costa Rica, which are geographically close to the type locality of *H*. *dulcensis*, are genetically distinct from those of *S*. *tsachila*.

## Discussion

Our examination of populations of *Scinax* from the Pacific basin of Ecuador demonstrates the existence of two distinct species masked under “*S*. *quinquefasciatus*”. In addition to their morphological similarity, both species have similar habitat preferences because they are frequently found in artificial open areas. Their elevation ranges are the only known ecological difference. *Scinax tsachila* has an elevational range twice as wide as that of *S*. *quinquefasciatus* (0–1207 m vs. 0–620 m).

Records of “*S*. *elaeochroa*” along Pacific basin of Colombia are spread from south to north [62] and need to be individually reassessed. *Scinax elaeochroa* is definitely known from the Caribbean lowlands of Nicaragua, Costa Rica, and extreme western Panama. Extensive fieldwork in eastern Panama has not revealed the existence of *S*. *elaeochroa*; consequently, specimens from the Pacific basin of Colombia referred to that species are more likely to be *S quinquefasciatus*, *S*, *tsachila*, *S*. *caprarius*, or an unnamed member of the genus.

The lowlands of western Ecuador are part of a biodiversity hotspot and, as such, have high concentration of endemic species and rapid habitat loss [67]. The Chocoan Rainforests and deciduous forests of Western Ecuador have the lowest proportion of remaining natural vegetation among natural habitats in Ecuador [68] and a high deforestation rate (2.2% per year; [59]). Habitat destruction is the most significant threat to amphibians worldwide [69] but, paradoxically, the increase in deforested areas should benefit both *S*. *quinquefasciatus* and *S*. *tsachila* because both species thrive in artificial open areas. Increase in size of distribution range and abundance is an unusual trend among Neotropical amphibians, a group having 39% of its species threatened with extinction [70]. Studies on the ecophysiology and general ecology of *S*. *quinquefasciatus* and *S*. *tsachila* could help to understand key adaptations of these amphibians to cope with anthropogenic habitat change.

## Supporting information

S1 Appendix Examined specimens of *Scinax*. (DOCX)
