# Supplementary material for: Advertisement calls and DNA sequences reveal a new species of Scinax (Anura: Hylidae) on the Pacific lowlands of Ecuador
Source: PLoS One. 2018 Sep 26;13(9):e0203169. doi: 10.1371/journal.pone.0203169 (PMC6157856; doi:10.1371/journal.pone.0203169)
Supplement: S1 Appendix — (DOCX) [file pone.0203169.s001.docx]

**S1 Appendix. Examined specimens of *Scinax*.** Asterisks indicate sequenced specimens. Abbreviations are: EC = Ecuador, CSR = Costa Rica, NP = National Park, PF = Protected Forest.

| **Museum No.** | **Species** | **Country** | **Province** | **Locality** |
| --- | --- | --- | --- | --- |
| QCAZ 206 | *S. cruentommus* | EC | Sucumbíos | Santa Cecilia, 340 m; 0.0805°N, 76.9906° W |
| QCAZ 442 | *S. cruentommus* | EC | Orellana | Puerto Francisco de Orellana (El Coca), 255 m; 0.4691° S, 76.9863° W |
| QCAZ 3901 | *S. cruentommus* | EC | Napo | Tena, 508 m; 0.9888° S, 77.8123° W |
| QCAZ 37882 | *S. cruentommus* | EC | Sucumbíos | Sacha Lodge, 237 m; 0.4712° S, 76.4590° W |
| QCAZ 39419 | *S. cruentommus* | EC | Pastaza | Bataburo Lodge, Cononaco road, 220 m; 1.2083° S, 76.7166° W |
| QCAZ 43829 | *S. cruentommus* | EC | Orellana | El Descanso, La Primavera, Río Napo, 244 m; 0.4442° S, 76.7868° W |
| QCAZ 44405 | *S. cruentommus* | EC | Orellana | Chiru Isla, Río Napo, 203 m; 0.5756° S, 75.8998° W |
| QCAZ 44752 | *S. cruentommus* | EC | Orellana | Santa Teresita, 4 km NW Nuevo Rocafuerte, Río Napo, 186 m; 0.9008° S, 75.4135° W |
| QCAZ 52531 | *S. cruentommus* | EC | Morona Santiago | Payapas-Santiago road, km 83, 315 m; 3.0467° S, 78.0132° W |
| QCAZ 52748 | *S. cruentommus* | EC | Pastaza | Sarayacu, Río Palandayacu, 365 m; 1.7371° S, 77.4935° W |
| QCAZ 53565 | *S. cruentommus* | EC | Pastaza | Juyuintza, 200 m; 2.11° S, 76.19° W |
| QCAZ 64167 | *S. cruentommus* | EC | Orellana | Yasuní NP, Río Yasuní, 223 m; 0.9069° S, 76.1446° W |
| QCAZ 67163 | *S. cruentommus* | EC | Zamora Chinchipe | Reserva Natural Maycu, 949 m; 4.3241° S, 78.6537° W |
| KU 30338 | *S. elaeochroa* | CSR | Cartago | Turrialba, 576 m; 9.8936°N, 83.6521° W |
| KU 30339 | *S. elaeochroa* | CSR | Cartago | Turrialba, 576 m; 9.8936°N, 83.6521° W |
| KU 30340 | *S. elaeochroa* | CSR | Cartago | Turrialba, 576 m; 9.8936°N, 83.6521° W |
| KU 30341 | *S. elaeochroa* | CSR | Cartago | Turrialba, 576 m; 9.8936°N, 83.6521° W |
| KU 30342 | *S. elaeochroa* | CSR | Cartago | Turrialba, 576 m; 9.8936°N, 83.6521° W |
| KU 30343 | *S. elaeochroa* | CSR | Cartago | Turrialba, 576 m; 9.8936°N, 83.6521° W |
| KU 30344 | *S. elaeochroa* | CSR | Cartago | Turrialba, 576 m; 9.8936°N, 83.6521° W |
| KU 30345 | *S. elaeochroa* | CSR | Cartago | Turrialba, 576 m; 9.8936°N, 83.6521° W |
| KU 30346 | *S. elaeochroa* | CSR | Cartago | Turrialba, 576 m; 9.8936°N, 83.6521° W |
| KU 30347 | *S. elaeochroa* | CSR | Cartago | Turrialba, 576 m; 9.8936°N, 83.6521° W |
| KU 30348 | *S. elaeochroa* | CSR | Cartago | Turrialba, 576 m; 9.8936°N, 83.6521° W |
| KU 30349 | *S. elaeochroa* | CSR | Cartago | Turrialba, 576 m; 9.8936°N, 83.6521° W |
| KU 30350 | *S. elaeochroa* | CSR | Cartago | Turrialba, 576 m; 9.8936°N, 83.6521° W |
| KU 30351 | *S. elaeochroa* | CSR | Cartago | Turrialba, 576 m; 9.8936°N, 83.6521° W |
| KU 30352 | *S. elaeochroa* | CSR | Cartago | Turrialba, 576 m; 9.8936°N, 83.6521° W |
| KU 30353 | *S. elaeochroa* | CSR | Cartago | Turrialba, 576 m; 9.8936°N, 83.6521° W |
| KU 30354 | *S. elaeochroa* | CSR | Cartago | Turrialba, 576 m; 9.8936°N, 83.6521° W |
| QCAZ 195 | *S. funereus* | EC | Sucumbíos | Santa Cecilia, 340 m; 0.0805°N, 76.9906° W |
| QCAZ 6555 | *S. funereus* | EC | Sucumbíos | San Pablo de Kantesiya, 243 m; 0.2505° S, 76.4255° W |
| QCAZ 25049 | *S. funereus* | EC | Orellana | Reserva Biológica Limoncocha, 246 m; 0.4079° S, 76.6206° W |
| QCAZ 25749 | *S. funereus* | EC | Napo | Inner Vision Lodge, Río Arajuno, 398 m; 1.1033° S, 77.5925° W |
| QCAZ 30941 | *S. funereus* | EC | Napo | Huino, 275 m; 0.6463° S, 77.1474° W |
| QCAZ 39444* | *S. funereus* | EC | Pastaza | Conambo, Bataburo Lodge, 220 m; 1.2083° S, 76.7166° W |
| QCAZ 43799* | *S. funereus* | EC | Orellana | El Descanso, La Primavera, Río Napo, 244 m; 0.4442° S, 76.7868° W |
| QCAZ 51043* | *S. funereus* | EC | Napo | 200 m W Estación Científica Jatun Sacha, 404 m; 1.0669° S, 77.602° W |
| QCAZ 52903 | *S. funereus* | EC | Pastaza | Comunidad Killu Allpa, 270 m; 2.1887° S, 76.8506° W |
| QCAZ 53123 | *S. funereus* | EC | Pastaza | Comunidad Campus, 354 m; 1.9810° S, 76.9162° W |
| QCAZ 53157 | *S. funereus* | EC | Pastaza | Comunidad Campus, 392 m; 1.9941° S, 76.9170° W |
| QCAZ 53260 | *S. funereus* | EC | Morona Santiago | Tukupi, 239 m; 2.7938° S, 77.4834° W |
| QCAZ 53929 | *S. funereus* | EC | Napo | Wildsumaco Wildlife Sanctuary, Wildsumaco Biological Station, 1450 m; 0.6761° S, 77.5993° W |
| QCAZ 55003 | *S. funereus* | EC | Pastaza | Villano camp (AGIP), km 26, 519 m; 1.4680° S, 77.6387° W |
| QCAZ 55283 | *S. funereus* | EC | Orellana | Yasuní NP, Tambococha, 191 m; 0.9736° S, 75.4352° W |
| QCAZ 57921 | *S. funereus* | EC | Orellana | San Vicente, Río Napo, 149 m; 0.6804° S, 75.7224° W |
| QCAZ 61779 | *S. funereus* | EC | Sucumbíos | Reserva Biológica Limoncocha, 258 m; 0.4004° S, 76.6237° W |
| QCAZ 61782 | *S. funereus* | EC | Sucumbíos | Reserva Biológica Limoncocha, 258 m; 0.4004° S, 76.6237° W |
| QCAZ 61784 | *S. funereus* | EC | Sucumbíos | Reserva Biológica Limoncocha, 258 m; 0.4004° S, 76.6237° W |
| QCAZ 64169 | *S. funereus* | EC | Orellana | Yasuní NP, Río Yasuní, 228 m; 0.9047° S, 76.1440° W |
| QCAZ 4775 | *S. garbei* | EC | Sucumbíos | Laguna Imuya, 178 m; 0.5831° S, 75.2508° W |
| QCAZ 16029 | *S. garbei* | EC | Zamora Chinchipe | Tundayme, 813 m; 3.5652° S, 78.4683° W |
| QCAZ 17450 | *S. garbei* | EC | Pastaza | Canelos, 524 m; 1.5883° S, 77.7736° W |
| QCAZ 25141 | *S. garbei* | EC | Orellana | Yasuní NP, Estación Científica Yasuní PUCE, 250 m; 0.6771° S, 76.4011° W |
| QCAZ 27257 | *S. garbei* | EC | Morona Santiago | Zamora, 927 m; 4.0491° S, 78.9256° W |
| QCAZ 32493 | *S. garbei* | EC | Morona Santiago | 900 m S Bobonaza, 660 m; 1.4980° S, 77.8793° W |
| QCAZ 37789 | *S. garbei* | EC | Sucumbíos | Reserva de Producción Faunística Cuyabeno, Laguna Grande, Cuyabeno Lodge, 213 m; 0.0096° S, 76.1817° W |
| QCAZ 40866 | *S. garbei* | EC | Pastaza | Centro Ecológico Zanjarajuno, 940 m; 1.3532° S, 77.8645° W |
| QCAZ 43098 | *S. garbei* | EC | Sucumbíos | Reserva Biológica Limoncocha, 261 m; 0.4062° S, 76.6194° W |
| QCAZ 43755* | *S. garbei* | EC | Orellana | 5 km S Puerto Francisco de Orellana (Coca), near Río Napo, 261 m; 0.4922° S, 77.0032° W |
| QCAZ 44478 | *S. garbei* | EC | Orellana | San Vicente, Río Napo, 196 m; 0.6790° S, 75.6511° W |
| QCAZ 44479 | *S. garbei* | EC | Orellana | San Vicente, Río Napo, 196 m; 0.6790° S, 75.6511° W |
| QCAZ 44597 | *S. garbei* | EC | Orellana | Santa María de Huiririma, Río Napo, 194 m; 0.7116° S, 75.6239° W |
| QCAZ 46403* | *S. garbei* | EC | Morona Santiago | Napimias, 543 m; 2.8177° S, 77.9987° W |
| QCAZ 47137 | *S. garbei* | EC | Napo | Comunidad Ita, 703 m; 0.9146° S, 77.7515° W |
| QCAZ 48844* | *S. garbei* | EC | Napo | Reserva Ecológica Yachana, 317 m; 0.8457° S, 77.2287° W |
| QCAZ 51223 | *S. garbei* | EC | Orellana | Yasuní NP, NPF-Tivacuno road, 234 m; 0.6876° S, 76.4290° W |
| QCAZ 55255 | *S. garbei* | EC | Orellana | Yasuní NP, Tambococha, 177 m; 0.9783° S, 75.4256° W |
| QCAZ 61789 | *S. garbei* | EC | Sucumbíos | Reserva Biológica Limoncocha, Laguna Limoncocha, 242 m; 0.4067° S, 76.6195° W |
| QCAZ 63017 | *S. garbei* | EC | Zamora Chinchipe | Concesión Mirador ECSA, San Marcos, near río Quimi, 801 m; 3.5727° S, 78.4671° W |
| QCAZ 64173 | *S. garbei* | EC | Orellana | Yasuní NP, Río Yasuní, Río Rumiyacu, 207 m; 0.9189° S, 75.8910° W |
| QCAZ 64972 | *S. garbei* | EC | Pastaza | Reserva Zanjarajuno, 937 m; 1.3713° S, 77.8536° W |
| KU 218471 | *S. quinquefasciatus* | EC | Guayas | 11.7 km S Milagro, 17 m; 2.221° S, 79.5699° W |
| KU 218472 | *S. quinquefasciatus* | EC | Guayas | 11.7 km S Milagro, 17 m; 2.221° S, 79.5699° W |
| KU 218477 | *S. quinquefasciatus* | EC | Guayas | 11.7 km S Milagro, 17 m; 2.221° S, 79.5699° W |
| KU 218483 | *S. quinquefasciatus* | EC | Los Ríos | 6.9 km N Babohoyo, 8 m; 1.739° S, 79.5319° W |
| KU 218486 | *S. quinquefasciatus* | EC | Los Ríos | 6.9 km N Babohoyo, 8 m; 1.739° S, 79.5319° W |
| KU 218488 | *S. quinquefasciatus* | EC | Los Ríos | 6.9 km N Babohoyo, 8 m; 1.739° S, 79.5319° W |
| QCAZ 2340 | *S. quinquefasciatus* | EC | Guayas | 11.7 km S Milagro, 50 m; 2.238° S, 79.643° W |
| QCAZ 2341 | *S. quinquefasciatus* | EC | Guayas | 11.7 km S Milagro, 50 m; 2.238° S, 79.643° W |
| QCAZ 2342 | *S. quinquefasciatus* | EC | Guayas | 11.7 km S Milagro, 50 m; 2.238° S, 79.643° W |
| QCAZ 2343 | *S. quinquefasciatus* | EC | Guayas | 11.7 km S Milagro, 50 m; 2.238° S, 79.643° W |
| QCAZ 2344 | *S. quinquefasciatus* | EC | Guayas | 11.7 km S Milagro, 50 m; 2.238° S, 79.643° W |
| QCAZ 2345 | *S. quinquefasciatus* | EC | Guayas | 11.7 km S Milagro, 50 m; 2.238° S, 79.643° W |
| QCAZ 2346 | *S. quinquefasciatus* | EC | Guayas | 11.7 km S Milagro, 50 m; 2.238° S, 79.643° W |
| QCAZ 2347 | *S. quinquefasciatus* | EC | Guayas | 11.7 km S Milagro, 50 m; 2.238° S, 79.643° W |
| QCAZ 2348 | *S. quinquefasciatus* | EC | Guayas | 11.7 km S Milagro, 50 m; 2.238° S, 79.643° W |
| QCAZ 2349 | *S. quinquefasciatus* | EC | Guayas | 11.7 km S Milagro, 50 m; 2.238° S, 79.643° W |
| QCAZ 2350 | *S. quinquefasciatus* | EC | Guayas | 11.7 km S Milagro, 50 m; 2.238° S, 79.643° W |
| QCAZ 2352 | *S. quinquefasciatus* | EC | Guayas | 11.7 km S Milagro, 50 m; 2.238° S, 79.643° W |
| QCAZ 2353 | *S. quinquefasciatus* | EC | Guayas | 11.7 km S Milagro, 50 m; 2.238° S, 79.643° W |
| QCAZ 2354 | *S. quinquefasciatus* | EC | Guayas | 11.7 km S Milagro, 50 m; 2.238° S, 79.643° W |
| QCAZ 2355 | *S. quinquefasciatus* | EC | Guayas | 11.7 km S Milagro, 50 m; 2.238° S, 79.643° W |
| QCAZ 2356 | *S. quinquefasciatus* | EC | Guayas | 11.7 km S Milagro, 50 m; 2.238° S, 79.643° W |
| QCAZ 2357 | *S. quinquefasciatus* | EC | Guayas | 11.7 km S Milagro, 50 m; 2.238° S, 79.643° W |
| QCAZ 2358 | *S. quinquefasciatus* | EC | Guayas | 11.7 km S Milagro, 50 m; 2.238° S, 79.643° W |
| QCAZ 2359 | *S. quinquefasciatus* | EC | Guayas | 11.7 km S Milagro, 50 m; 2.238° S, 79.643° W |
| QCAZ 2561 | *S. quinquefasciatus* | EC | Guayas | Guayaquil, 9 m; 2.1666° S, 79.9° W |
| QCAZ 4195 | *S. quinquefasciatus* | EC | Los Ríos | 8 km S Ventanas, 18 m; 1.515° S, 79.4689° W |
| QCAZ 4196 | *S. quinquefasciatus* | EC | Los Ríos | 8 km S Ventanas, 18 m; 1.515° S, 79.4689° W |
| QCAZ 4197 | *S. quinquefasciatus* | EC | Los Ríos | 8 km S Ventanas, 18 m; 1.515° S, 79.4689° W |
| QCAZ 4559 | *S. quinquefasciatus* | EC | Esmeraldas | Viche, Estación Refugio Silvestre, 60 m; 0.6615°N, 79.5387° W |
| QCAZ 4628 | *S. quinquefasciatus* | EC | Esmeraldas | Same, 10 m; 0.8428°N, 79.9217° W |
| QCAZ 4629 | *S. quinquefasciatus* | EC | Esmeraldas | Same, 22 m; 0.8428°N, 79.9217° W |
| QCAZ 4630 | *S. quinquefasciatus* | EC | Esmeraldas | Same, 10 m; 0.8428°N, 79.9217° W |
| QCAZ 4631 | *S. quinquefasciatus* | EC | Esmeraldas | Same, 10 m; 0.8428°N, 79.9217° W |
| QCAZ 7260 | *S. quinquefasciatus* | EC | Esmeraldas | Durango, 202 m; 1.0427°N, 78.6244° W |
| QCAZ 7262 | *S. quinquefasciatus* | EC | Esmeraldas | Durango, 202 m; 1.0427°N, 78.6244° W |
| QCAZ 8478 | *S. quinquefasciatus* | EC | Esmeraldas | Same, 10 m; 0.8428°N, 79.9217° W |
| QCAZ 8563 | *S. quinquefasciatus* | EC | Manabí | El Aromo, 309 m; 1.0466° S, 80.8327° W |
| QCAZ 10158 | *S. quinquefasciatus* | EC | Manabí | 17.5 km Cabo Pasado, San Vicente-Pedernales road, 50 m; 0.3689° S, 80.489° W |
| QCAZ 12624 | *S. quinquefasciatus* | EC | Cañar | Manta Real, 500 m; 2.5536° S, 79.3642° W |
| QCAZ 12625 | *S. quinquefasciatus* | EC | Cañar | Manta Real, 500 m; 2.5536° S, 79.3642° W |
| QCAZ 12784 | *S. quinquefasciatus* | EC | Cañar | Manta Real, 500 m; 2.5536° S, 79.3642° W |
| QCAZ 12785 | *S. quinquefasciatus* | EC | Cañar | Manta Real, 500 m; 2.5536° S, 79.3642° W |
| QCAZ 12790 | *S. quinquefasciatus* | EC | Cañar | Manta Real, 500 m; 2.5536° S, 79.3642° W |
| QCAZ 12791 | *S. quinquefasciatus* | EC | Cañar | Manta Real, 500 m; 2.5536° S, 79.3642° W |
| QCAZ 12792 | *S. quinquefasciatus* | EC | Cañar | Manta Real, 500 m; 2.5536° S, 79.3642° W |
| QCAZ 12793 | *S. quinquefasciatus* | EC | Cañar | Manta Real, 500 m; 2.5536° S, 79.3642° W |
| QCAZ 12803 | *S. quinquefasciatus* | EC | Cañar | Manta Real, 500 m; 2.5536° S, 79.3642° W |
| QCAZ 13915 | *S. quinquefasciatus* | EC | Cañar | Manta Real, 265 m; 2.5569° S, 79.369° W |
| QCAZ 15005 | *S. quinquefasciatus* | EC | Esmeraldas | Río Verde, 12 m; 1.0741°N, 79.4091° W |
| QCAZ 15006 | *S. quinquefasciatus* | EC | Esmeraldas | Río Verde, 12 m; 1.0741°N, 79.4091° W |
| QCAZ 16994 | *S. quinquefasciatus* | EC | El Oro | Portovelo, 635 m; 3.7541° S, 79.64861° W |
| QCAZ 17120 | *S. quinquefasciatus* | EC | El Oro | Río Moromoro, 924 m; 3.6638° S, 79.7372° W |
| QCAZ 18595 | *S. quinquefasciatus* | EC | Los Ríos | 6.9 km N Babohoyo, 60 m; 1.738° S, 79.533° W |
| QCAZ 19368 | *S. quinquefasciatus* | EC | Guayas | Cerro Más Vale, 50 m; 2.3970° S, 79.6323° W |
| QCAZ 19921 | *S. quinquefasciatus* | EC | Manabí | Puerto Rico, 15 m; 1.6392° S, 80.8303° W |
| QCAZ 19922 | *S. quinquefasciatus* | EC | Guayas | Cerro Más Vale, 58 m; 2.3959° S, 79.6301° W |
| QCAZ 19923 | *S. quinquefasciatus* | EC | Guayas | Cerro Más Vale, 58 m; 2.3959° S, 79.6301° W |
| QCAZ 19924 | *S. quinquefasciatus* | EC | Guayas | Cerro Más Vale, 58 m; 2.3959° S, 79.6301° W |
| QCAZ 19925 | *S. quinquefasciatus* | EC | Guayas | Cerro Más Vale, 58 m; 2.3959° S, 79.6301° W |
| QCAZ 19926 | *S. quinquefasciatus* | EC | Guayas | Cerro Más Vale, 58 m; 2.3959° S, 79.6301° W |
| QCAZ 19927 | *S. quinquefasciatus* | EC | Guayas | Cerro Más Vale, 58 m; 2.3959° S, 79.6301° W |
| QCAZ 19928 | *S. quinquefasciatus* | EC | Guayas | Cerro Más Vale, 58 m; 2.3959° S, 79.6301° W |
| QCAZ 19929 | *S. quinquefasciatus* | EC | Guayas | Cerro Más Vale, 58 m; 2.3959° S, 79.6301° W |
| QCAZ 21000 | *S. quinquefasciatus* | EC | Guayas | 11.7 km S Milagro, 50 m; 2.238° S, 79.643° W |
| QCAZ 21949 | *S. quinquefasciatus* | EC | Guayas | 11.7 km S Milagro, 11 m; 2.238° S, 79.643° W |
| QCAZ 21950 | *S. quinquefasciatus* | EC | Guayas | Milagro, 11 m; 2.1322° S, 79.5891° W |
| QCAZ 21951 | *S. quinquefasciatus* | EC | Guayas | Milagro, 11 m; 2.1322° S, 79.5891° W |
| QCAZ 21952 | *S. quinquefasciatus* | EC | Guayas | Milagro, 11 m; 2.1322° S, 79.5891° W |
| QCAZ 22306 | *S. quinquefasciatus* | EC | Esmeraldas | La Tola, 31 m; 1.2119°N, 79.0434° W |
| QCAZ 22741 | *S. quinquefasciatus* | EC | Esmeraldas | La Tola, 31 m; 1.2119°N, 79.0434° W |
| QCAZ 22906 | *S. quinquefasciatus* | EC | Guayas | 11.7 km S Milagro, 50 m; 2.238° S, 79.643° W |
| QCAZ 23177 | *S. quinquefasciatus* | EC | Manabí | 20 km NW El Carmen, Pedernales road, 184 m; 0.1472° S, 79.5656° W |
| QCAZ 23178 | *S. quinquefasciatus* | EC | Manabí | 20 km NW El Carmen, Pedernales road, 184 m; 0.1472° S, 79.5656° W |
| QCAZ 23179 | *S. quinquefasciatus* | EC | Manabí | 20 km NW El Carmen, Pedernales road, 184 m; 0.1472° S, 79.5656° W |
| QCAZ 23180 | *S. quinquefasciatus* | EC | Manabí | 20 km NW El Carmen, Pedernales road, 184 m; 0.1472° S, 79.5656° W |
| QCAZ 23181 | *S. quinquefasciatus* | EC | Manabí | 20 km NW El Carmen, Pedernales road, 184 m; 0.1472° S, 79.5656° W |
| QCAZ 23182 | *S. quinquefasciatus* | EC | Manabí | 20 km NW El Carmen, Pedernales road, 184 m; 0.1472° S, 79.5656° W |
| QCAZ 23346 | *S. quinquefasciatus* | EC | Esmeraldas | 20 km E Pedernales, El Carmen road, 113 m; 0.0024°N, 79.8961° W |
| QCAZ 23347 | *S. quinquefasciatus* | EC | Esmeraldas | 20 km E Pedernales, El Carmen road, 113 m; 0.0024°N, 79.8961° W |
| QCAZ 23348 | *S. quinquefasciatus* | EC | Esmeraldas | 20 km E Pedernales, El Carmen road, 113 m; 0.0024°N, 79.8961° W |
| QCAZ 23349 | *S. quinquefasciatus* | EC | Esmeraldas | 20 km E Pedernales, El Carmen road, 113 m; 0.0024°N, 79.8961° W |
| QCAZ 23360 | *S. quinquefasciatus* | EC | Esmeraldas | 20 km E Pedernales, El Carmen road, 113 m; 0.0024°N, 79.8961° W |
| QCAZ 23378 | *S. quinquefasciatus* | EC | Guayas | Las Palmas-Balsas road, 58 m; 2.0165° S, 80.4960° W |
| QCAZ 23382 | *S. quinquefasciatus* | EC | Guayas | Las Palmas-Balsas road, 58 m; 2.0165° S, 80.4960° W |
| QCAZ 23383 | *S. quinquefasciatus* | EC | Guayas | Las Palmas-Balsas road, 58 m; 2.0165° S, 80.4960° W |
| QCAZ 23384 | *S. quinquefasciatus* | EC | Guayas | Las Palmas-Balsas road, 58 m; 2.0165° S, 80.4960° W |
| QCAZ 23385 | *S. quinquefasciatus* | EC | Guayas | Las Palmas-Balsas road, 58 m; 2.0165° S, 80.4960° W |
| QCAZ 23398* | *S. quinquefasciatus* | EC | Guayas | El Palmar-Balsas road, 50 m; 2.0078° S, 80.6037° W |
| QCAZ 23451* | *S. quinquefasciatus* | EC | Guayas | 20 km E Durán, Milagro road, 32 m; 2.0222° S, 79.6905° W |
| QCAZ 23452 | *S. quinquefasciatus* | EC | Guayas | 20 km E Durán, Milagro road, 32 m; 2.0222° S, 79.6905° W |
| QCAZ 23453 | *S. quinquefasciatus* | EC | Guayas | 20 km E Durán, Milagro road, 32 m; 2.0222° S, 79.6905° W |
| QCAZ 23454 | *S. quinquefasciatus* | EC | Guayas | 20 km E Durán, Milagro road, 32 m; 2.0222° S, 79.6905° W |
| QCAZ 23455 | *S. quinquefasciatus* | EC | Guayas | 20 km E Durán, Milagro road, 32 m; 2.0222° S, 79.6905° W |
| QCAZ 23456 | *S. quinquefasciatus* | EC | Guayas | 20 km E Durán, Milagro road, 32 m; 2.0222° S, 79.6905° W |
| QCAZ 23468 | *S. quinquefasciatus* | EC | Guayas | Estación Cerro Más Vale, 147 m; 2.3986° S, 79.63349° W |
| QCAZ 23478 | *S. quinquefasciatus* | EC | Guayas | Cerro Más Vale, 147 m; 2.3986° S, 79.6334° W |
| QCAZ 23538 | *S. quinquefasciatus* | EC | Guayas | 11 Km N Cerro Más Vale, Virgen de Fátima road, 40 m; 2.3003° S, 79.6388° W |
| QCAZ 23539* | *S. quinquefasciatus* | EC | Guayas | 11 Km N Cerro Más Vale, Virgen de Fátima road, 40 m; 2.3003° S, 79.6388° W |
| QCAZ 23593 | *S. quinquefasciatus* | EC | Guayas | 15 km Naranjal, Machala road, 26 m; 2.7658° S, 79.6918° W |
| QCAZ 23658 | *S. quinquefasciatus* | EC | Guayas | Naranjal-El Guabo road, 38 m; 2.8235° S, 79.7008° W |
| QCAZ 23659 | *S. quinquefasciatus* | EC | Guayas | Naranjal-El Guabo road, 38 m; 2.8235° S, 79.7008° W |
| QCAZ 23660 | *S. quinquefasciatus* | EC | Guayas | Naranjal-El Guabo road, 38 m; 2.8235° S, 79.7008° W |
| QCAZ 23679 | *S. quinquefasciatus* | EC | El Oro | Arenillas, Huaquillas-La Cuca road, 53 m; 3.5356° S, 80.0671° W |
| QCAZ 23689 | *S. quinquefasciatus* | EC | El Oro | Arenillas, Huaquillas-La Cuca road, 53 m; 3.5356° S, 80.0671° W |
| QCAZ 23763 | *S. quinquefasciatus* | EC | El Oro | Puyango PF, 295 m; 3.8819° S, 80.0830° W |
| QCAZ 23764 | *S. quinquefasciatus* | EC | El Oro | Puyango PF, 295 m; 3.8819° S, 80.0830° W |
| QCAZ 23950 | *S. quinquefasciatus* | EC | Galápagos | Puerto Villamil, Poza de las Diablas, 5 m; 0.9509° S, 90.9782° W |
| QCAZ 23951 | *S. quinquefasciatus* | EC | Galápagos | Puerto Villamil, Poza de las Diablas, 5 m; 0.9509° S, 90.9782° W |
| QCAZ 23952 | *S. quinquefasciatus* | EC | Galápagos | Puerto Villamil, Poza de las Diablas, 5 m; 0.9509° S, 90.9782° W |
| QCAZ 23953 | *S. quinquefasciatus* | EC | Galápagos | Puerto Villamil, Poza de las Diablas, 5 m; 0.9509° S, 90.9782° W |
| QCAZ 23954 | *S. quinquefasciatus* | EC | Galápagos | Puerto Villamil, Poza de las Diablas, 5 m; 0.9509° S, 90.9782° W |
| QCAZ 23956 | *S. quinquefasciatus* | EC | Galápagos | Puerto Villamil, Poza de las Diablas, 5 m; 0.9509° S, 90.9782° W |
| QCAZ 23957 | *S. quinquefasciatus* | EC | Galápagos | Puerto Villamil, Poza de las Diablas, 5 m; 0.9509° S, 90.9782° W |
| QCAZ 23958 | *S. quinquefasciatus* | EC | Galápagos | Puerto Villamil, Poza de las Diablas, 5 m; 0.9509° S, 90.9782° W |
| QCAZ 23959 | *S. quinquefasciatus* | EC | Galápagos | Puerto Villamil, Poza de las Diablas, 5 m; 0.9509° S, 90.9782° W |
| QCAZ 23960 | *S. quinquefasciatus* | EC | Galápagos | Puerto Villamil, Poza de las Diablas, 5 m; 0.9509° S, 90.9782° W |
| QCAZ 23961 | *S. quinquefasciatus* | EC | Galápagos | Puerto Villamil, Poza de las Diablas, 5 m; 0.9509° S, 90.9782° W |
| QCAZ 23962 | *S. quinquefasciatus* | EC | Galápagos | Puerto Villamil, Poza de las Diablas, 5 m; 0.9509° S, 90.9782° W |
| QCAZ 23963 | *S. quinquefasciatus* | EC | Galápagos | Puerto Villamil, Poza de las Diablas, 5 m; 0.9509° S, 90.9782° W |
| QCAZ 23964 | *S. quinquefasciatus* | EC | Galápagos | Puerto Villamil, Poza de las Diablas, 5 m; 0.9509° S, 90.9782° W |
| QCAZ 23965 | *S. quinquefasciatus* | EC | Galápagos | Puerto Villamil, Poza de las Diablas, 5 m; 0.9509° S, 90.9782° W |
| QCAZ 23966 | *S. quinquefasciatus* | EC | Galápagos | Puerto Villamil, Poza de las Diablas, 5 m; 0.9509° S, 90.9782° W |
| QCAZ 23967 | *S. quinquefasciatus* | EC | Galápagos | Puerto Villamil, Poza de las Diablas, 5 m; 0.9509° S, 90.9782° W |
| QCAZ 23968 | *S. quinquefasciatus* | EC | Galápagos | Puerto Villamil, Poza de las Diablas, 5 m; 0.9509° S, 90.9782° W |
| QCAZ 23973 | *S. quinquefasciatus* | EC | Pichincha | 6 km NW Pedro Vicente Maldonado, 209 m; 0.1042°N, 79.1027° W |
| QCAZ 23974 | *S. quinquefasciatus* | EC | Pichincha | 6 km NW Pedro Vicente Maldonado, 209 m; 0.1042°N, 79.1027° W |
| QCAZ 23992 | *S. quinquefasciatus* | EC | Guayas | Cerro Blanco PF, 300 m; 2.1783° S, 80.0213° W |
| QCAZ 24965 | *S. quinquefasciatus* | EC | Esmeraldas | Borbon, 97 m; 1.0792°N, 79.0174° W |
| QCAZ 24966 | *S. quinquefasciatus* | EC | Esmeraldas | Borbon, 97 m; 1.0792°N, 79.0174° W |
| QCAZ 26068 | *S. quinquefasciatus* | EC | Manabí | Estero Ancho, 329 m; 0.0687° S, 79.7963° W |
| QCAZ 26792 | *S. quinquefasciatus* | EC | Esmeraldas | 1 km S Esmeraldas, Atacames road, 10 m; 0.9304°N, 79.6765° W |
| QCAZ 26794 | *S. quinquefasciatus* | EC | Esmeraldas | 1 km S Esmeraldas, Esmeraldas-Atacames road, 10 m; 0.9304°N, 79.6765° W |
| QCAZ 26795 | *S. quinquefasciatus* | EC | Esmeraldas | 1 km S Esmeraldas, Esmeraldas-Atacames road, 10 m; 0.9304°N, 79.6765° W |
| QCAZ 26796 | *S. quinquefasciatus* | EC | Esmeraldas | 1 km S Esmeraldas, Esmeraldas-Atacames road, 10 m; 0.9304°N, 79.6765° W |
| QCAZ 26797 | *S. quinquefasciatus* | EC | Esmeraldas | 1 km S Esmeraldas, Esmeraldas-Atacames road, 10 m; 0.9304°N, 79.6765° W |
| QCAZ 26801 | *S. quinquefasciatus* | EC | Esmeraldas | Súa, 1 m; 0.8589°N, 79.8755° W |
| QCAZ 26802 | *S. quinquefasciatus* | EC | Esmeraldas | Súa, 1 m; 0.8589°N, 79.8755° W |
| QCAZ 26830 | *S. quinquefasciatus* | EC | Guayas | Quevedo, El Empalme road, 64 m; 1.0642° S, 79.4917° W |
| QCAZ 26832 | *S. quinquefasciatus* | EC | Guayas | Quevedo, El Empalme road, 64 m; 1.0642° S, 79.4917° W |
| QCAZ 26833 | *S. quinquefasciatus* | EC | Guayas | Quevedo, El Empalme road, 64 m; 1.0642° S, 79.4917° W |
| QCAZ 26834 | *S. quinquefasciatus* | EC | Guayas | Quevedo, El Empalme road, 64 m; 1.0642° S, 79.4917° W |
| QCAZ 26835 | *S. quinquefasciatus* | EC | Guayas | Quevedo, El Empalme road, 64 m; 1.0642° S, 79.4917° W |
| QCAZ 26940* | *S. quinquefasciatus* | EC | Guayas | 1 km S Milagro, Naranjal road, 7 m; 2.1530° S, 79.6027° W |
| QCAZ 27018 | *S. quinquefasciatus* | EC | El Oro | 1 km Río Puyango, Alamor-Arenillas road, Puyango PF, 325 m; 3.8802° S, 80.0655° W |
| QCAZ 27019 | *S. quinquefasciatus* | EC | El Oro | Puyango PF, 1 km Río Puyango, Alamor-Arenillas road, 325 m; 3.8802° S, 80.0655° W |
| QCAZ 27636 | *S. quinquefasciatus* | EC | Guayas | 11.7 km S Milagro, 50 m; 2.2431° S, 79.6432° W |
| QCAZ 29231 | *S. quinquefasciatus* | EC | Guayas | Cerro Más Vale, 17 m; 2.3959° S, 79.6301° W |
| QCAZ 29232 | *S. quinquefasciatus* | EC | Guayas | Cerro Más Vale, 17 m; 2.3959° S, 79.6301° W |
| QCAZ 29233 | *S. quinquefasciatus* | EC | Guayas | Cerro Más Vale, 17 m; 2.3959° S, 79.6301° W |
| QCAZ 29234 | *S. quinquefasciatus* | EC | Guayas | Cerro Más Vale, 17 m; 2.3959° S, 79.6301° W |
| QCAZ 29235 | *S. quinquefasciatus* | EC | Guayas | Cerro Más Vale, 17 m; 2.3959° S, 79.6301° W |
| QCAZ 29236 | *S. quinquefasciatus* | EC | Guayas | Cerro Más Vale, 17 m; 2.3959° S, 79.6301° W |
| QCAZ 29237 | *S. quinquefasciatus* | EC | Guayas | Cerro Más Vale, 17 m; 2.3959° S, 79.6301° W |
| QCAZ 29238 | *S. quinquefasciatus* | EC | Guayas | Cerro Más Vale, 17 m; 2.3959° S, 79.6301° W |
| QCAZ 29239 | *S. quinquefasciatus* | EC | Guayas | Cerro Más Vale, 17 m; 2.3959° S, 79.6301° W |
| QCAZ 29610 | *S. quinquefasciatus* | EC | Guayas | Cerro Más Vale, Guayaquil-Puerto Inca road, 17 m; 2.3959° S, 79.63017° W |
| QCAZ 29611 | *S. quinquefasciatus* | EC | Guayas | Cerro Más Vale, Guayaquil-Puerto Inca road, 17 m; 2.3959° S, 79.63017° W |
| QCAZ 29612 | *S. quinquefasciatus* | EC | Guayas | Cerro Más Vale, Guayaquil-Puerto Inca road, 17 m; 2.3959° S, 79.63017° W |
| QCAZ 29613 | *S. quinquefasciatus* | EC | Guayas | Cerro Más Vale, Guayaquil-Puerto Inca road, 17 m; 2.3959° S, 79.63017° W |
| QCAZ 29614 | *S. quinquefasciatus* | EC | Guayas | Cerro Más Vale, Guayaquil-Puerto Inca road, 17 m; 2.3959° S, 79.63017° W |
| QCAZ 29615 | *S. quinquefasciatus* | EC | Guayas | Cerro Más Vale, Guayaquil-Puerto Inca road, 17 m; 2.3959° S, 79.6301° W |
| QCAZ 29616 | *S. quinquefasciatus* | EC | Guayas | Cerro Más Vale, Guayaquil-Puerto Inca road, 17 m; 2.3959° S, 79.6301° W |
| QCAZ 30201 | *S. quinquefasciatus* | EC | Manabí | El Carmen, Chone road, km 36, 300 m; 0.2737° S, 79.4586° W |
| QCAZ 30596 | *S. quinquefasciatus* | EC | El Oro | La Avanzada-Playón road, 230 m; 3.581° S, 79.938° W |
| QCAZ 31542 | *S. quinquefasciatus* | EC | Los Ríos | Juan Montalvo, Río Cristal, 65 m; 1.7940° S, 79.2843° W |
| QCAZ 35892 | *S. quinquefasciatus* | EC | Galápagos | Puerto Villamil, Poza de las Diablas, 5 m; 0.9569° S, 90.9782° W |
| QCAZ 39867 | *S. quinquefasciatus* | EC | El Oro | 3 km Huaquillas, Arenillas road, 23 m; 3.5236° S, 80.2069° W |
| QCAZ 39868 | *S. quinquefasciatus* | EC | El Oro | 3 km Huaquillas, Arenillas road, 23 m; 3.5236° S, 80.2069° W |
| QCAZ 40227 | *S. quinquefasciatus* | EC | Guayas | Marcelino Maridueña, Ingenio San Carlos, 26 m; 2.2126° S, 79.4471° W |
| QCAZ 40499 | *S. quinquefasciatus* | EC | Galápagos | Isabela, 1503 m; 0.9287° S, 91.4044° W |
| QCAZ 41411 | *S. quinquefasciatus* | EC | Guayas | Cerro Blanco PF, 237 m; 2.18° S, 80.0197° W |
| QCAZ 41451 | *S. quinquefasciatus* | EC | Guayas | Guayaquil, km 24, 73 m; 2.253° S, 80.144° W |
| QCAZ 42228 | *S. quinquefasciatus* | EC | Manabí | Descanso de los Romeros, 153 m; 0.4047° S, 79.9057° W |
| QCAZ 42254 | *S. quinquefasciatus* | EC | Los Ríos | Centro Cientifico Río Palenque, 271 m; 0.5666° S, 79.3333° W |
| QCAZ 42255 | *S. quinquefasciatus* | EC | Los Ríos | Centro Cientifico Río Palenque, 271 m; 0.5666° S, 79.3333° W |
| QCAZ 46488 | *S. quinquefasciatus* | EC | Santo Domingo de los Tsáchilas | La Concordia, La Perla PF, 190 m; 0.057° S, 79.3589° W |
| QCAZ 46788 | *S. quinquefasciatus* | EC | Galápagos | Poza de las Diablas, 8 m; 0.9465° S, 90.9783° W |
| QCAZ 46789 | *S. quinquefasciatus* | EC | Galápagos | Poza de las Diablas, 8 m; 0.9465° S, 90.9783° W |
| QCAZ 46790 | *S. quinquefasciatus* | EC | Galápagos | Poza de las Diablas, 8 m; 0.9465° S, 90.9783° W |
| QCAZ 46791 | *S. quinquefasciatus* | EC | Galápagos | Poza de las Diablas, 8 m; 0.9465° S, 90.9783° W |
| QCAZ 46792 | *S. quinquefasciatus* | EC | Galápagos | Poza de las Diablas, 8 m; 0.9465° S, 90.9783° W |
| QCAZ 46793 | *S. quinquefasciatus* | EC | Galápagos | Poza de las Diablas, 8 m; 0.9465° S, 90.9783° W |
| QCAZ 46794 | *S. quinquefasciatus* | EC | Galápagos | Poza de las Diablas, 8 m; 0.9465° S, 90.9783° W |
| QCAZ 46795 | *S. quinquefasciatus* | EC | Galápagos | Poza de las Diablas, 8 m; 0.9465° S, 90.9783° W |
| QCAZ 46796 | *S. quinquefasciatus* | EC | Galápagos | Poza de las Diablas, 8 m; 0.9465° S, 90.9783° W |
| QCAZ 46797 | *S. quinquefasciatus* | EC | Galápagos | Poza de las Diablas, 8 m; 0.9465° S, 90.9783° W |
| QCAZ 46798 | *S. quinquefasciatus* | EC | Galápagos | Poza de las Diablas, 8 m; 0.9465° S, 90.9783° W |
| QCAZ 46799 | *S. quinquefasciatus* | EC | Galápagos | Poza de las Diablas, 8 m; 0.9465° S, 90.9783° W |
| QCAZ 46800 | *S. quinquefasciatus* | EC | Galápagos | Poza de las Diablas, 8 m; 0.9465° S, 90.9783° W |
| QCAZ 48732 | *S. quinquefasciatus* | EC | Manabí | Río Manta, 165 m; 1.075° S, 80.7369° W |
| QCAZ 49972 | *S. quinquefasciatus* | EC | Esmeraldas | Playa escondida, 30 m; 0.8177°N, 80.0032° W |
| QCAZ 50671 | *S. quinquefasciatus* | EC | Manabí | Río Coaque, 33 m; 0.0501°N, 80.0388° W |
| QCAZ 50672 | *S. quinquefasciatus* | EC | Manabí | Río Coaque, 33 m; 0.0501°N, 80.0388° W |
| QCAZ 50677 | *S. quinquefasciatus* | EC | Manabí | Río Coaque-Jama road, 11 m; 0.0566°N, 80.0584° W |
| QCAZ 50679 | *S. quinquefasciatus* | EC | Manabí | Río Coaque-Jama road, 11 m; 0.0566°N, 80.0584° W |
| QCAZ 50680 | *S. quinquefasciatus* | EC | Manabí | Río Coaque-Jama road, 11 m; 0.0566°N, 80.0584° W |
| QCAZ 50681 | *S. quinquefasciatus* | EC | Manabí | Río Coaque-Jama road, 1 m; 0.0464° S, 80.1400° W |
| QCAZ 50685 | *S. quinquefasciatus* | EC | Manabí | Río Coaque-Jama road, 1 m; 0.0464° S, 80.1400° W |
| QCAZ 50686 | *S. quinquefasciatus* | EC | Manabí | Río Coaque-Jama road, 1 m; 0.0464° S, 80.1400° W |
| QCAZ 50691 | *S. quinquefasciatus* | EC | Manabí | Río Coaque-Jama road, 1 m; 0.0464° S, 80.1400° W |
| QCAZ 50704 | *S. quinquefasciatus* | EC | Manabí | 5 km N Rocafuerte, San Andrés de Rocafuerte, 334 m; 0.8682° S, 80.4636° W |
| QCAZ 50707 | *S. quinquefasciatus* | EC | Manabí | 5 km N Rocafuerte, San Andrés de Rocafuerte, 334 m; 0.8682° S, 80.4636° W |
| QCAZ 50708 | *S. quinquefasciatus* | EC | Manabí | 5 km N Rocafuerte, San Andrés de Rocafuerte, 334 m; 0.8682° S, 80.4636° W |
| QCAZ 50710 | *S. quinquefasciatus* | EC | Manabí | 5 km N Rocafuerte, San Andrés de Rocafuerte, 334 m; 0.8682° S, 80.4636° W |
| QCAZ 50711 | *S. quinquefasciatus* | EC | Manabí | 5 km N Rocafuerte, San Andrés de Rocafuerte, 334 m; 0.8682° S, 80.4636° W |
| QCAZ 50712 | *S. quinquefasciatus* | EC | Manabí | 5 km N Rocafuerte, San Andrés de Rocafuerte, 334 m; 0.8682° S, 80.4636° W |
| QCAZ 50713 | *S. quinquefasciatus* | EC | Manabí | 5 km N Rocafuerte, San Andrés de Rocafuerte, 334 m; 0.8682° S, 80.4636° W |
| QCAZ 50714 | *S. quinquefasciatus* | EC | Manabí | 5 km N Rocafuerte, San Andrés de Rocafuerte, 334 m; 0.8682° S, 80.4636° W |
| QCAZ 51491 | *S. quinquefasciatus* | EC | Los Ríos | Reserva Ecológica Pedro Franco Dávila, Recinto Jauneche, 36 m; 1.2466° S, 79.6601° W |
| QCAZ 55637 | *S. quinquefasciatus* | EC | El Oro | Huaquillas, 15 m; 3.4091° S, 79.9471° W |
| QCAZ 58052 | *S. quinquefasciatus* | EC | El Oro | Arenillas, 55 m; 3.5667° S, 80.1432° W |
| QCAZ 60994 | *S. quinquefasciatus* | EC | Guayas | Balzar, 43 m; 1.1755° S, 79.9644° W |
| QCAZ 61938 | *S. quinquefasciatus* | EC | El Oro | Reserva Ecologica Arenillas, 35 m; 3.5766° S, 80.1210° W |
| QCAZ 61939 | *S. quinquefasciatus* | EC | El Oro | Reserva Ecológica Arenillas, 3 m; 3.5109° S, 80.1231° W |
| QCAZ 62975 | *S. quinquefasciatus* | EC | El Oro | Zaracay-Piñas road, 735 m; 3.6623° S, 79.7621° W |
| QCAZ 63434 | *S. quinquefasciatus* | EC | El Oro | 1 km Río Puyango, Alamor-Arenillas road, Puyango PF, 325 m; 3.8802° S, 80.0655° W |
| QCAZ 70362 | *S. quinquefasciatus* | EC | Guayas | San Pablo, 16 m; 2.5711° S, 80.3657° W |
| QCAZ 70363 | *S. quinquefasciatus* | EC | Guayas | San Pablo, 16 m; 2.5711° S, 80.3657° W |
| QCAZ 70364 | *S. quinquefasciatus* | EC | Guayas | San Pablo, 16 m; 2.5711° S, 80.3657° W |
| QCAZ 70365 | *S. quinquefasciatus* | EC | Guayas | San Pablo, 16 m; 2.5711° S, 80.3657° W |
| QCAZ 70366 | *S. quinquefasciatus* | EC | Guayas | San Pablo, 16 m; 2.5711° S, 80.3657° W |
| QCAZ 70367 | *S. quinquefasciatus* | EC | Guayas | San Pablo, 16 m; 2.5711° S, 80.3657° W |
| QCAZ 70368 | *S. quinquefasciatus* | EC | Guayas | San Pablo, 16 m; 2.5711° S, 80.3657° W |
| QCAZ 70369 | *S. quinquefasciatus* | EC | Guayas | San Pablo, 16 m; 2.5711° S, 80.3657° W |
| QCAZ 70370 | *S. quinquefasciatus* | EC | Guayas | San Pablo, 16 m; 2.5711° S, 80.3657° W |
| QCAZ 70371 | *S. quinquefasciatus* | EC | Guayas | San Pablo, 16 m; 2.5711° S, 80.3657° W |
| QCAZ 70372 | *S. quinquefasciatus* | EC | Guayas | San Pablo, 16 m; 2.5711° S, 80.3657° W |
| QCAZ 70373 | *S. quinquefasciatus* | EC | Guayas | San Pablo, 16 m; 2.5711° S, 80.3657° W |
| QCAZ 70374 | *S. quinquefasciatus* | EC | Guayas | San Pablo, 16 m; 2.5711° S, 80.3657° W |
| QCAZ 70375 | *S. quinquefasciatus* | EC | Guayas | San Pablo, 16 m; 2.5711° S, 80.3657° W |
| QCAZ 70376 | *S. quinquefasciatus* | EC | Guayas | San Pablo, 16 m; 2.5711° S, 80.3657° W |
| QCAZ 70377 | *S. quinquefasciatus* | EC | Guayas | San Pablo, 16 m; 2.5711° S, 80.3657° W |
| QCAZ 70378 | *S. quinquefasciatus* | EC | Guayas | San Pablo, 16 m; 2.5711° S, 80.3657° W |
| QCAZ 70379 | *S. quinquefasciatus* | EC | Guayas | San Pablo, 16 m; 2.5711° S, 80.3657° W |
| QCAZ 70380 | *S. quinquefasciatus* | EC | Guayas | San Pablo, 16 m; 2.5711° S, 80.3657° W |
| QCAZ 70381 | *S. quinquefasciatus* | EC | Guayas | San Pablo, 16 m; 2.5711° S, 80.3657° W |
| QCAZ 70382 | *S. quinquefasciatus* | EC | Guayas | San Pablo, 16 m; 2.5711° S, 80.3657° W |
| QCAZ 70383 | *S. quinquefasciatus* | EC | Guayas | San Pablo, 16 m; 2.5711° S, 80.3657° W |
| QCAZ 70384 | *S. quinquefasciatus* | EC | Guayas | San Pablo, 16 m; 2.5711° S, 80.3657° W |
| QCAZ 70385 | *S. quinquefasciatus* | EC | Guayas | San Pablo, 16 m; 2.5711° S, 80.3657° W |
| QCAZ 70386 | *S. quinquefasciatus* | EC | Guayas | San Pablo, 16 m; 2.5711° S, 80.3657° W |
| QCAZ 70387 | *S. quinquefasciatus* | EC | Guayas | San Pablo, 16 m; 2.5711° S, 80.3657° W |
| QCAZ 70388 | *S. quinquefasciatus* | EC | Guayas | San Pablo, 16 m; 2.5711° S, 80.3657° W |
| QCAZ 70389 | *S. quinquefasciatus* | EC | Guayas | San Pablo, 16 m; 2.5711° S, 80.3657° W |
| QCAZ 70390 | *S. quinquefasciatus* | EC | Guayas | San Pablo, 16 m; 2.5711° S, 80.3657° W |
| QCAZ 70391 | *S. quinquefasciatus* | EC | Guayas | San Pablo, 16 m; 2.5711° S, 80.3657° W |
| QCAZ 70392 | *S. quinquefasciatus* | EC | Guayas | San Pablo, 16 m; 2.5711° S, 80.3657° W |
| QCAZ 70393 | *S. quinquefasciatus* | EC | Guayas | San Pablo, 16 m; 2.5711° S, 80.3657° W |
| QCAZ 70394 | *S. quinquefasciatus* | EC | Guayas | San Pablo, 16 m; 2.5711° S, 80.3657° W |
| QCAZ 70395 | *S. quinquefasciatus* | EC | Guayas | San Pablo, 16 m; 2.5711° S, 80.3657° W |
| QCAZ 70396 | *S. quinquefasciatus* | EC | Guayas | San Pablo, 16 m; 2.5711° S, 80.3657° W |
| QCAZ 70397 | *S. quinquefasciatus* | EC | Guayas | San Pablo, 30 m; 2.5694° S, 80.3920° W |
| QCAZ 70398 | *S. quinquefasciatus* | EC | Guayas | San Pablo, 30 m; 2.5694° S, 80.3920° W |
| QCAZ 70399 | *S. quinquefasciatus* | EC | Guayas | San Pablo, 30 m; 2.5694° S, 80.3920° W |
| QCAZ 70400 | *S. quinquefasciatus* | EC | Guayas | San Pablo, 30 m; 2.5694° S, 80.3920° W |
| QCAZ 70401 | *S. quinquefasciatus* | EC | Guayas | Área Nacional de Recreación Parque Lago, Represa Chongón, 45 m; 2.2143° S, 80.1042° W |
| QCAZ 70402 | *S. quinquefasciatus* | EC | Guayas | Área Nacional de Recreación Parque Lago, Represa Chongón, 45 m; 2.2143° S, 80.1042° W |
| QCAZ 70403 | *S. quinquefasciatus* | EC | Guayas | Área Nacional de Recreación Parque Lago, Represa Chongón, 51 m; 2.2142° S, 80.1047° W |
| QCAZ 70404 | *S. quinquefasciatus* | EC | Guayas | Área Nacional de Recreación Parque Lago, Represa Chongón, 55 m; 2.2149° S, 80.1048° W |
| QCAZ 70405 | *S. quinquefasciatus* | EC | Guayas | Área Nacional de Recreación Parque Lago, Represa Chongón, 45 m; 2.2143° S, 80.1042° W |
| QCAZ 70406 | *S. quinquefasciatus* | EC | Guayas | Área Nacional de Recreación Parque Lago, Represa Chongón, 45 m; 2.2143° S, 80.1042° W |
| QCAZ 70407 | *S. quinquefasciatus* | EC | Guayas | Área Nacional de Recreación Parque Lago, Represa Chongón, 45 m; 2.2143° S, 80.1042° W |
| QCAZ 70408 | *S. quinquefasciatus* | EC | Guayas | Área Nacional de Recreación Parque Lago, Represa Chongón, 49 m; 2.2153° S, 80.1050° W |
| QCAZ 70409 | *S. quinquefasciatus* | EC | Guayas | Área Nacional de Recreación Parque Lago, Represa Chongón, 49 m; 2.2153° S, 80.1050° W |
| QCAZ 1005 | *S. ruber* | EC | Morona Santiago | 53.8 km E Bella Unión, Santiago road, 1010 m; 3.0329° S, 78.14° W |
| QCAZ 10205 | *S. ruber* | EC | Orellana | Yasuní NP, Estación Biológica Tiputini USFQ, 250 m; 0.6387° S, 76.1492° W |
| QCAZ 10258 | *S. ruber* | EC | Sucumbíos | 5 km E Lumbaqui, 850 m; 0.062°N, 77.2939° W |
| QCAZ 10310 | *S. ruber* | EC | Sucumbíos | 5 km E Lumbaqui, 850 m; 0.0179°N, 77.3229° W |
| QCAZ 10383 | *S. ruber* | EC | Sucumbíos | Lumbaqui-La Bonita road, km 1, 700 m; 0.0502°N, 77.3222° W |
| QCAZ 10384 | *S. ruber* | EC | Sucumbíos | Lumbaqui-La Bonita road, km 1, 700 m; 0.0502°N, 77.3222° W |
| QCAZ 10388 | *S. ruber* | EC | Sucumbíos | Montañas de Lumbaqui, 6 Km E Lumbaqui, 850 m; 0.0092°N, 77.3218° W |
| QCAZ 10389 | *S. ruber* | EC | Sucumbíos | Montañas de Lumbaqui, 6 Km E Lumbaqui, 850 m; 0.0092°N, 77.3218° W |
| QCAZ 10398 | *S. ruber* | EC | Sucumbíos | Puerto Libre, 700 m; 0.1912°N, 77.4882° W |
| QCAZ 10412 | *S. ruber* | EC | Sucumbíos | Sinangue, Río Candué, 650 m; 0.0859°N, 77.388° W |
| QCAZ 43729* | *S. ruber* | EC | Orellana | 5 km S Puerto Francisco de Orellana (Coca), near Río Napo, 257 m; 0.4850° S, 76.9848° W |
| QCAZ 51062* | *S. ruber* | EC | Orellana | Yasuní NP, Estación Científica Yasuní PUCE, 218 m; 0.6743° S, 76.3970° W |
| KU 146444 | *S. sugillatus* | EC | Los Ríos | Estación Biológica Río Palenque, 56 km N Quevedo, 220 m; 0.55° S, 79.3666° W |
| KU 146445 | *S. sugillatus* | EC | Los Ríos | Estación Biológica Río Palenque, 56 km N Quevedo, 220 m; 0.55° S, 79.3666° W |
| KU 146446 | *S. sugillatus* | EC | Los Ríos | Estación Biológica Río Palenque, 56 km N Quevedo, 220 m; 0.55° S, 79.3666° W |
| KU 146447 | *S. sugillatus* | EC | Los Ríos | Estación Biológica Río Palenque, 56 km N Quevedo, 220 m; 0.55° S, 79.3666° W |
| KU 146448 | *S. sugillatus* | EC | Los Ríos | Estación Biológica Río Palenque, 56 km N Quevedo, 220 m; 0.55° S, 79.3666° W |
| KU 146449 | *S. sugillatus* | EC | Los Ríos | Estación Biológica Río Palenque, 56 km N Quevedo, 220 m; 0.55° S, 79.3666° W |
| QCAZ 296 | *S. sugillatus* | EC | Santo Domingo de los Tsáchilas | La Concordia, La Perla PF, 257 m; 0.057° S, 79.3589° W |
| QCAZ 297 | *S. sugillatus* | EC | Santo Domingo de los Tsáchilas | La Concordia, La Perla PF, 257 m; 0.057° S, 79.3589° W |
| QCAZ 437 | *S. sugillatus* | EC | Los Ríos | Centro Cientifico Río Palenque, 271 m; 0.5666° S, 79.3333° W |
| QCAZ 787 | *S. sugillatus* | EC | Esmeraldas | La Unión, 94 m; 0.762°N, 79.4609° W |
| QCAZ 2351 | *S. sugillatus* | EC | Guayas | 11.7 km S Milagro, 50 m; 2.238° S, 79.643° W |
| QCAZ 5009 | *S. sugillatus* | EC | Esmeraldas | Viche, 36 m; 0.6615°N, 79.5387° W |
| QCAZ 6331 | *S. sugillatus* | EC | Esmeraldas | Viche, Granja Fauna Tropical, 200 m; 0.6615°N, 79.5387° W |
| QCAZ 6408 | *S. sugillatus* | EC | Esmeraldas | Viche, Granja de Profauna, 36 m; 0.6615°N, 79.5387° W |
| QCAZ 21032 | *S. sugillatus* | EC | Santo Domingo de los Tsáchilas | 6 km W La Concordia, 190 m; 0.003°N, 79.45° W |
| QCAZ 21033 | *S. sugillatus* | EC | Santo Domingo de los Tsáchilas | 6 km W La Concordia, 190 m; 0.003°N, 79.45° W |
| QCAZ 21034 | *S. sugillatus* | EC | Santo Domingo de los Tsáchilas | 6 km W La Concordia, 190 m; 0.003°N, 79.45° W |
| QCAZ 22889 | *S. sugillatus* | EC | Santo Domingo de los Tsáchilas | 6 km La Concordia, 190 m; 0.0219° S, 79.361° W |
| QCAZ 27219 | *S. sugillatus* | EC | Esmeraldas | Boca de Sabateta, 101 m; 0.3118°N, 79.5988° W |
| QCAZ 27220 | *S. sugillatus* | EC | Esmeraldas | Boca de Sabateta, 101 m; 0.3118°N, 79.5988° W |
| QCAZ 27632 | *S. sugillatus* | EC | Santo Domingo de los Tsáchilas | Santo Domingo de los Tsáchilas, 6 km W La Concordia, 190 m; 0.2569° S, 79.1661° W |
| QCAZ 31529 | *S. sugillatus* | EC | Esmeraldas | San Lorenzo road, Río Bogotá, 37 m; 1.1805°N, 78.7536° W |
| QCAZ 33998 | *S. sugillatus* | EC | Santo Domingo de los Tsáchilas | La Concordia, La Perla PF, 190 m; 0.057° S, 79.3589° W |
| QCAZ 34102 | *S. sugillatus* | EC | Santo Domingo de los Tsáchilas | La Concordia, La Perla PF, 190 m; 0.057° S, 79.3589° W |
| QCAZ 34112 | *S. sugillatus* | EC | Santo Domingo de los Tsáchilas | La Concordia, La Perla PF, 190 m; 0.057° S, 79.3589° W |
| QCAZ 35275 | *S. sugillatus* | EC | Los Ríos | Buena Fé, Recinto Corriente Grande, La Isla, 127 m; 0.6727° S, 79.3950° W |
| QCAZ 35276 | *S. sugillatus* | EC | Los Ríos | Buena Fé, Recinto Corriente Grande, La Isla, 127 m; 0.6727° S, 79.3950° W |
| QCAZ 35277 | *S. sugillatus* | EC | Los Ríos | Buena Fé, Recinto Corriente Grande, La Isla, 127 m; 0.6727° S, 79.3950° W |
| QCAZ 35279 | *S. sugillatus* | EC | Los Ríos | Buena Fé, Recinto Corriente Grande, La Isla, 127 m; 0.6727° S, 79.3950° W |
| QCAZ 35280 | *S. sugillatus* | EC | Los Ríos | Buena Fé, Recinto Corriente Grande, La Isla, 127 m; 0.6727° S, 79.3950° W |
| QCAZ 35706 | *S. sugillatus* | EC | Esmeraldas | La Mayronga, 150 m; 1.05°N, 79.27° W |
| QCAZ 35707 | *S. sugillatus* | EC | Esmeraldas | La Mayronga, 150 m; 1.05°N, 79.27° W |
| QCAZ 35708 | *S. sugillatus* | EC | Esmeraldas | La Mayronga, 150 m; 1.05°N, 79.27° W |
| QCAZ 40336 | *S. sugillatus* | EC | Esmeraldas | Rosa Zarate, Laguna del Cube, 207 m; 0.4150°N, 79.6496° W |
| QCAZ 40337 | *S. sugillatus* | EC | Esmeraldas | Rosa Zarate, Laguna del Cube, 207 m; 0.4150°N, 79.6496° W |
| QCAZ 40338 | *S. sugillatus* | EC | Esmeraldas | Rosa Zarate, Laguna del Cube, 207 m; 0.4150°N, 79.6496° W |
| QCAZ 40339 | *S. sugillatus* | EC | Esmeraldas | Rosa Zarate, Laguna del Cube, 207 m; 0.4150°N, 79.6496° W |
| QCAZ 40340 | *S. sugillatus* | EC | Esmeraldas | Rosa Zarate, Laguna del Cube, 207 m; 0.4150°N, 79.6496° W |
| QCAZ 40341 | *S. sugillatus* | EC | Esmeraldas | Rosa Zarate, Laguna del Cube, 207 m; 0.4150°N, 79.6496° W |
| QCAZ 40353 | *S. sugillatus* | EC | Esmeraldas | Rosa Zarate, Laguna del Cube, 352 m; 0.3916°N, 79.6479° W |
|  |  | EC |  |  |
| QCAZ 47085 | *S. sugillatus* | EC | Pichincha | Centro Cientifico Río Palenque, 195 m; 0.5888° S, 79.3627° W |
| QCAZ 55525 | *S. sugillatus* | EC | Esmeraldas | Durango, 188 m; 1.0427°N, 78.6244° W |
| QCAZ 57783 | *S. sugillatus* | EC | Esmeraldas | Durango, 214 m; 1.0408°N, 78.6247° W |
| QCAZ 65691 | *S. sugillatus* | EC | Esmeraldas | Reserva Tesoro Escondido, 454 m; 0.5355°N, 79.1508° W |
| QCAZ 66649 | *S. sugillatus* | EC | Esmeraldas | Durango, 20 km SE Tundaloma Lodge, 192 m; 1.0369°N, 78.6267° W |
| KU218492* | *S. tsachila* | EC | Pichincha | 1 km E Vicente Maldonado, 613 m; 0.0859°N, 79.043° W |
| KU218493* | *S. tsachila* | EC | Pichincha | 1 km E Vicente Maldonado, 613 m; 0.0859°N, 79.043° W |
| KU218494* | *S. tsachila* | EC | Pichincha | 1 km E Vicente Maldonado, 613 m; 0.0859°N, 79.043° W |
| KU218495* | *S. tsachila* | EC | Pichincha | 1 km E Vicente Maldonado, 613 m; 0.0859°N, 79.043° W |
| KU218498* | *S. tsachila* | EC | Pichincha | 1 km E Vicente Maldonado, 613 m; 0.0859°N, 79.043° W |
| KU218503* | *S. tsachila* | EC | Pichincha | 1 km W La Concordia, 212 m; 0.0089°N, 79.396° W |
| KU218504* | *S. tsachila* | EC | Pichincha | 1 km W La Concordia, 212 m; 0.0089°N, 79.396° W |
| KU218505* | *S. tsachila* | EC | Pichincha | 1 km W La Concordia, 212 m; 0.0089°N, 79.396° W |
| QCAZ 179 | *S. tsachila* | EC | Los Ríos | Río Palenque, 158 m; 0.5865° S, 79.3647° W |
| QCAZ 432 | *S. tsachila* | EC | Pichincha | Puerto Quito, 193 m; 0.1261°N, 79.2524° W |
| QCAZ 783 | *S. tsachila* | EC | Santo Domingo de los Tsáchilas | La Concordia, 297 m; 0.0666° S, 79.3166° W |
| QCAZ 784 | *S. tsachila* | EC | Santo Domingo de los Tsáchilas | Quinindé road, km 29, 700 m; 0.069° S, 79.3259° W |
| QCAZ 2295 | *S. tsachila* | EC | Santo Domingo de los Tsáchilas | Santo Domingo, 552 m; 0.2569° S, 79.1661° W |
| QCAZ 3510 | *S. tsachila* | EC | Santo Domingo de los Tsáchilas | La Perla PF, 5 km E La Concordia, 190 m; 0.057° S, 79.3589° W |
| QCAZ 3511 | *S. tsachila* | EC | Santo Domingo de los Tsáchilas | La Perla PF, 5 km E La Concordia, 190 m; 0.057° S, 79.3589° W |
| QCAZ 3512 | *S. tsachila* | EC | Santo Domingo de los Tsáchilas | La Perla PF, 5 km E La Concordia, 190 m; 0.057° S, 79.3589° W |
| QCAZ 3748 | *S. tsachila* | EC | Pichincha | 1 km E Pedro Vicente Maldonado, 745 m; 0.0832°N, 79.0394° W |
| QCAZ 3775 | *S. tsachila* | EC | Pichincha | 1 km E Maldonado, 745 m; 0.0832°N, 79.0394° W |
| QCAZ 3776 | *S. tsachila* | EC | Pichincha | 1 km E Maldonado, 745 m; 0.0832°N, 79.0394° W |
| QCAZ 3777 | *S. tsachila* | EC | Pichincha | 1 km E Maldonado, 745 m; 0.0832°N, 79.0394° W |
| QCAZ 3778 | *S. tsachila* | EC | Pichincha | 1 km E Maldonado, 745 m; 0.0832°N, 79.0394° W |
| QCAZ 3779 | *S. tsachila* | EC | Pichincha | 1 km E Maldonado, 745 m; 0.0832°N, 79.0394° W |
| QCAZ 3780 | *S. tsachila* | EC | Pichincha | 1 km E Maldonado, 745 m; 0.0832°N, 79.0394° W |
| QCAZ 3868 | *S. tsachila* | EC | Pichincha | San Miguel de los Bancos-Puerto Quito road, km 9, 819 m; 0.0720°N, 78.9599° W |
| QCAZ 6810 | *S. tsachila* | EC | Los Ríos | 2.4 km W Guasaganda, Río Quindigua, 350 m; 0.7855° S, 79.2731° W |
| QCAZ 6811 | *S. tsachila* | EC | Los Ríos | 2.4 km W Guasaganda, Río Quindigua, 350 m; 0.7855° S, 79.2731° W |
| QCAZ 6812 | *S. tsachila* | EC | Los Ríos | 2.4 km W Guasaganda, Río Quindigua, 350 m; 0.7855° S, 79.2731° W |
| QCAZ 6813 | *S. tsachila* | EC | Los Ríos | 2.4 km W Guasaganda, Río Quindigua, 350 m; 0.7855° S, 79.2731° W |
| QCAZ 6814 | *S. tsachila* | EC | Los Ríos | 2.4 km W Guasaganda, Río Quindigua, 350 m; 0.7855° S, 79.2731° W |
| QCAZ 6815 | *S. tsachila* | EC | Los Ríos | 2.4 km W Guasaganda, Río Quindigua, 350 m; 0.7855° S, 79.2731° W |
| QCAZ 6816 | *S. tsachila* | EC | Los Ríos | 2.4 km W Guasaganda, Río Quindigua, 350 m; 0.7855° S, 79.2731° W |
| QCAZ 6817 | *S. tsachila* | EC | Los Ríos | 2.4 km W Guasaganda, Río Quindigua, 350 m; 0.7855° S, 79.2731° W |
| QCAZ 6818 | *S. tsachila* | EC | Los Ríos | 2.4 km W Guasaganda, Río Quindigua, 350 m; 0.7855° S, 79.2731° W |
| QCAZ 6819 | *S. tsachila* | EC | Los Ríos | 2.4 km W Guasaganda, Río Quindigua, 350 m; 0.7855° S, 79.2731° W |
| QCAZ 6836 | *S. tsachila* | EC | Los Ríos | 2.4 km W Guasaganda, Río Quindigua, 350 m; 0.7855° S, 79.2731° W |
| QCAZ 6907 | *S. tsachila* | EC | Los Ríos | 2.4 km W Guasaganda, Río Quindigua, 350 m; 0.7855° S, 79.2731° W |
| QCAZ 7258 | *S. tsachila* | EC | Esmeraldas | Durango, 202 m; 1.0427°N, 78.6244° W |
| QCAZ 7259 | *S. tsachila* | EC | Esmeraldas | Durango, 202 m; 1.0427°N, 78.6244° W |
| QCAZ 7261 | *S. tsachila* | EC | Esmeraldas | Durango, 202 m; 1.0427°N, 78.6244° W |
| QCAZ 7861 | *S. tsachila* | EC | Pichincha | 1 km E Pedro Vicente Maldonado, 670 m; 0.0866°N, 79.0373° W |
| QCAZ 10283 | *S. tsachila* | EC | Esmeraldas | Corriente Grande, Río Cayapas, 70 m; 0.6895°N, 78.9589° W |
| QCAZ 10612 | *S. tsachila* | EC | Esmeraldas | Lita-San Lorenzo road, near La Boca, 22 m; 1.133°N, 78.774° W |
| QCAZ 10613 | *S. tsachila* | EC | Esmeraldas | Lita-San Lorenzo road, near La Boca, 22 m; 1.133°N, 78.774° W |
| QCAZ 10614 | *S. tsachila* | EC | Esmeraldas | Lita-San Lorenzo road, near La Boca, 22 m; 1.133°N, 78.774° W |
| QCAZ 10615 | *S. tsachila* | EC | Esmeraldas | Lita-San Lorenzo road, near La Boca, 22 m; 1.133°N, 78.774° W |
| QCAZ 10616 | *S. tsachila* | EC | Esmeraldas | Lita-San Lorenzo road, near La Boca, 22 m; 1.133°N, 78.774° W |
| QCAZ 11633 | *S. tsachila* | EC | Guayas | El Piedrero, 15 km E El Triunfo, Pallatanga road, 230 m; 2.3443° S, 79.3067° W |
| QCAZ 11634 | *S. tsachila* | EC | Guayas | El Piedrero, 15 km E El Triunfo, Pallatanga road, 230 m; 2.3443° S, 79.3067° W |
| QCAZ 11635 | *S. tsachila* | EC | Guayas | El Piedrero, 15 km E El Triunfo, Pallatanga road, 230 m; 2.3443° S, 79.3067° W |
| QCAZ 11636 | *S. tsachila* | EC | Guayas | El Piedrero, 15 km E El Triunfo, Pallatanga road, 230 m; 2.3443° S, 79.3067° W |
| QCAZ 11637 | *S. tsachila* | EC | Guayas | El Piedrero, 15 km E El Triunfo, Pallatanga road, 230 m; 2.3443° S, 79.3067° W |
| QCAZ 15541 | *S. tsachila* | EC | Esmeraldas | 3 km Durango, 158 m; 1.0643°N, 78.6443° W |
| QCAZ 15542 | *S. tsachila* | EC | Esmeraldas | 3 km Durango, 158 m; 1.0643°N, 78.6443° W |
| QCAZ 15543 | *S. tsachila* | EC | Esmeraldas | 3 km Durango, 158 m; 1.0643°N, 78.6443° W |
| QCAZ 15932 | *S. tsachila* | EC | Esmeraldas | 3 km Durango, Lita-San Lorenzo road, 158 m; 1.0643°N, 78.6443° W |
| QCAZ 17542 | *S. tsachila* | EC | Los Ríos | Río Palenque, 158 m; 0.5888° S, 79.3627° W |
| QCAZ 17543 | *S. tsachila* | EC | Los Ríos | Río Palenque, 158 m; 0.5888° S, 79.3627° W |
| QCAZ 17544 | *S. tsachila* | EC | Los Ríos | Río Palenque, 158 m; 0.5888° S, 79.3627° W |
| QCAZ 17545 | *S. tsachila* | EC | Los Ríos | Río Palenque, 158 m; 0.5888° S, 79.3627° W |
| QCAZ 17546 | *S. tsachila* | EC | Los Ríos | Río Palenque, 158 m; 0.5888° S, 79.3627° W |
| QCAZ 17547 | *S. tsachila* | EC | Los Ríos | Río Palenque, 158 m; 0.5888° S, 79.3627° W |
| QCAZ 17548 | *S. tsachila* | EC | Los Ríos | Río Palenque, 158 m; 0.5888° S, 79.3627° W |
| QCAZ 17549 | *S. tsachila* | EC | Los Ríos | Río Palenque, 175 m; 0.5888° S, 79.3627° W |
| QCAZ 18594 | *S. tsachila* | EC | Pichincha | 1 km W Vicente Maldonado, 670 m; 0.097°N, 79.0669° W |
| QCAZ 20106 | *S. tsachila* | EC | Pichincha | La Unión del Toachi, 915 m; 0.3138° S, 78.9544° W |
| QCAZ 20985 | *S. tsachila* | EC | Pichincha | 1 km E Pedro Vicente Maldonado, 635 m; 0.0832°N, 79.0394° W |
| QCAZ 21029 | *S. tsachila* | EC | Pichincha | 1 km E Pedro Vicente Maldonado, 635 m; 0.0832°N, 79.0394° W |
| QCAZ 21030 | *S. tsachila* | EC | Pichincha | 1 km E Pedro Vicente Maldonado, 635 m; 0.0832°N, 79.0394° W |
| QCAZ 21031 | *S. tsachila* | EC | Pichincha | 1 km E Pedro Vicente Maldonado, 635 m; 0.0832°N, 79.0394° W |
| QCAZ 22699 | *S. tsachila* | EC | Esmeraldas | 1 km E Pedro Vicente Maldonado, 635 m; 0.0832°N, 79.0394° W |
| QCAZ 22894 | *S. tsachila* | EC | Pichincha | 1 km E Pedro Vicente Maldonado, 635 m; 0.0832°N, 79.0394° W |
| QCAZ 22912 | *S. tsachila* | EC | Pichincha | 1 km E Pedro Vicente Maldonado, 635 m; 0.0832°N, 79.0394° W |
| QCAZ 23157 | *S. tsachila* | EC | Esmeraldas | Durango, 297 m; 1.0252°N, 78.5729° W |
| QCAZ 23173 | *S. tsachila* | EC | Pichincha | Nueva Israel, 340 m; 0.2437° S, 79.3435° W |
| QCAZ 23174 | *S. tsachila* | EC | Pichincha | Nueva Israel, 340 m; 0.2437° S, 79.3435° W |
| QCAZ 23175 | *S. tsachila* | EC | Pichincha | Nueva Israel, 340 m; 0.2437° S, 79.3435° W |
| QCAZ 23183* | *S. tsachila* | EC | Manabí | 20 km NW El Carmen, Pedernales road, 184 m; 0.1472° S, 79.5656° W |
| QCAZ 23184* | *S. tsachila* | EC | Manabí | Estero Ancho, 52 km W El Carmen, Pedernales road, 329 m; 0.0677° S, 79.7960° W |
| QCAZ 23185* | *S. tsachila* | EC | Manabí | Estero Ancho, 52 km W El Carmen, Pedernales road, 329 m; 0.0677° S, 79.7960° W |
| QCAZ 23479 | *S. tsachila* | EC | Guayas | Cerro Más Vale, 560 m; 2.3986° S, 79.6334° W |
| QCAZ 23554 | *S. tsachila* | EC | Esmeraldas | Durango, 346 m; 1.0252°N, 78.5729° W |
| QCAZ 23619 | *S. tsachila* | EC | Esmeraldas | 31.2 km E Viche, 120 m; 0.4083°N, 79.5229° W |
| QCAZ 23672* | *S. tsachila* | EC | El Oro | Arenillas, Huaquillas-La Cuca road, 53 m; 3.5356° S, 80.0671° W |
| QCAZ 23673* | *S. tsachila* | EC | El Oro | Arenillas, Huaquillas-La Cuca road, 53 m; 3.5356° S, 80.0671° W |
| QCAZ 23678* | *S. tsachila* | EC | El Oro | Arenillas, Huaquillas-La Cuca road, 53 m; 3.5356° S, 80.0671° W |
| QCAZ 23702 | *S. tsachila* | EC | El Oro | Arenillas, Huaquillas-La Cuca road, 53 m; 3.5356° S, 80.0671° W |
| QCAZ 24558 | *S. tsachila* | EC | Imbabura | Lita, 585 m; 0.8695°N, 78.4501° W |
| QCAZ 24559 | *S. tsachila* | EC | Imbabura | Lita, 585 m; 0.8695°N, 78.4501° W |
| QCAZ 24560 | *S. tsachila* | EC | Imbabura | Lita, 585 m; 0.8695°N, 78.4501° W |
| QCAZ 24561 | *S. tsachila* | EC | Imbabura | Lita, 585 m; 0.8695°N, 78.4501° W |
| QCAZ 24562 | *S. tsachila* | EC | Imbabura | Lita, 585 m; 0.8695°N, 78.4501° W |
| QCAZ 24613 | *S. tsachila* | EC | Esmeraldas | Quinindé, 83 m; 0.3316°N, 79.4675° W |
| QCAZ 26102 | *S. tsachila* | EC | Esmeraldas | Durango, 7 km San Lorenzo road, 74 m; 1.0793°N, 78.6695° W |
| QCAZ 26104 | *S. tsachila* | EC | Esmeraldas | Durango, 7 km San Lorenzo road, 74 m; 1.0793°N, 78.6695° W |
| QCAZ 27017 | *S. tsachila* | EC | El Oro | 1 km Río Puyango, Alamor-Arenillas road, Puyango PF, 325 m; 3.8802° S, 80.0655° W |
| QCAZ 27221 | *S. tsachila* | EC | Esmeraldas | Boca de Sabateta, 101 m; 0.3118°N, 79.5988° W |
| QCAZ 27626 | *S. tsachila* | EC | Pichincha | 1 km E Pedro Vicente Maldonado, 670 m; 0.0828°N, 79.0382° W |
| QCAZ 27627 | *S. tsachila* | EC | Pichincha | 1 km E Pedro Vicente Maldonado, 670 m; 0.0828°N, 79.0382° W |
| QCAZ 27628 | *S. tsachila* | EC | Pichincha | 1 km E Pedro Vicente Maldonado, 670 m; 0.0828°N, 79.0382° W |
| QCAZ 27629 | *S. tsachila* | EC | Pichincha | 1 km E Pedro Vicente Maldonado, 670 m; 0.0828°N, 79.0382° W |
| QCAZ 27630 | *S. tsachila* | EC | Pichincha | 1 km E Pedro Vicente Maldonado, 670 m; 0.0828°N, 79.0382° W |
| QCAZ 27631 | *S. tsachila* | EC | Pichincha | 1 km E Pedro Vicente Maldonado, 670 m; 0.0828°N, 79.0382° W |
| QCAZ 27633 | *S. tsachila* | EC | Santo Domingo de los Tsáchilas | Santo Domingo de los Tsáchilas, 5.3 km W La Concordia, 190 m; 0.2569° S, 79.1661° W |
| QCAZ 27634 | *S. tsachila* | EC | Santo Domingo de los Tsáchilas | Santo Domingo de los Tsáchilas, 1 km W La Concordia, 190 m; 0.2569° S, 79.1661° W |
| QCAZ 27668 | *S. tsachila* | EC | Pichincha | 1 km E Pedro Vicente Maldonado, 670 m; 0.0866°N, 79.0373° W |
| QCAZ 28529 | *S. tsachila* | EC | Santo Domingo de los Tsáchilas | Tinalandia, Río Toachi, 694 m; 0.3030° S, 79.0410° W |
| QCAZ 30760 | *S. tsachila* | EC | Imbabura | 3 km E Lita, 670 m; 0.8453°N, 78.4496° W |
| QCAZ 30761 | *S. tsachila* | EC | Imbabura | 3 km E Lita, 670 m; 0.8453°N, 78.4496° W |
| QCAZ 30762 | *S. tsachila* | EC | Imbabura | 3 km E Lita, 670 m; 0.8453°N, 78.4496° W |
| QCAZ 30763 | *S. tsachila* | EC | Imbabura | 3 km E Lita, 670 m; 0.8453°N, 78.4496° W |
| QCAZ 30764 | *S. tsachila* | EC | Imbabura | 3.8 km E Lita, 670 m; 0.8389°N, 78.4509° W |
| QCAZ 30765 | *S. tsachila* | EC | Imbabura | 3.8 km E Lita, 670 m; 0.8389°N, 78.4509° W |
| QCAZ 30766 | *S. tsachila* | EC | Imbabura | 3.8 km E Lita, 670 m; 0.8389°N, 78.4509° W |
| QCAZ 30767 | *S. tsachila* | EC | Imbabura | 3.8 km E Lita, 670 m; 0.8389°N, 78.4509° W |
| QCAZ 30768 | *S. tsachila* | EC | Imbabura | 3.8 km E Lita, 670 m; 0.8389°N, 78.4509° W |
| QCAZ 30769 | *S. tsachila* | EC | Imbabura | 3.8 km E Lita, 670 m; 0.8389°N, 78.4509° W |
| QCAZ 31757 | *S. tsachila* | EC | Cotopaxi | Naranjito, Bosque Integral Otonga, Las Damas, 1338 m; 0.3988° S, 78.9797° W |
| QCAZ 33473 | *S. tsachila* | EC | Manabí | Calceta, Palo Seco, 100 m; 0.8451° S, 80.1642° W |
| QCAZ 33474 | *S. tsachila* | EC | Manabí | Calceta, Palo Seco, 100 m; 0.8451° S, 80.1642° W |
| QCAZ 33999 | *S. tsachila* | EC | Santo Domingo de los Tsáchilas | La Concordia, La Perla PF, 190 m; 0.057° S, 79.3589° W |
| QCAZ 34101 | *S. tsachila* | EC | Santo Domingo de los Tsáchilas | La Concordia, La Perla PF, 190 m; 0.057° S, 79.3589° W |
| QCAZ 35714 | *S. tsachila* | EC | Esmeraldas | La Mayronga, 150 m; 1.05°N, 79.27° W |
| QCAZ 35715 | *S. tsachila* | EC | Esmeraldas | La Mayronga, 150 m; 1.05°N, 79.27° W |
| QCAZ 39880* | *S. tsachila* | EC | El Oro | La Avanzada, 4 km Santa Rosa, Las Balsas road, 44 m; 3.5142° S, 79.9659° W |
| QCAZ 40228 | *S. tsachila* | EC | Guayas | Marcelino Maridueña, Ingenio San Carlos, 26 m; 2.2126° S, 79.4471° W |
| QCAZ 40843* | *S. tsachila* | EC | Esmeraldas | Playón de San Francisco, 82 m; 1.0872°N, 78.6904° W |
| QCAZ 40848 | *S. tsachila* | EC | Esmeraldas | Playón de San Francisco, 82 m; 1.0872°N, 78.6904° W |
| QCAZ 40849 | *S. tsachila* | EC | Esmeraldas | Playón de San Francisco, 82 m; 1.0872°N, 78.6904° W |
| QCAZ 41416 | *S. tsachila* | EC | Guayas | Cerro Blanco PF, 237 m; 2.18° S, 80.0197° W |
| QCAZ 42289 | *S. tsachila* | EC | El Oro | 4 km Santa Rosa, La avanzada, Santa Rosa-Las Balsas road, 44 m; 3.5142° S, 79.9659° W |
| QCAZ 45267 | *S. tsachila* | EC | Santo Domingo de los Tsáchilas | 16 km E Santo Domingo de los Tsáchilas, 600 m; 0.2727° S, 79.079° W |
| QCAZ 45423* | *S. tsachila* | EC | Esmeraldas | Durango, 243 m; 1.0418°N, 78.6240° W |
| QCAZ 45424* | *S. tsachila* | EC | Esmeraldas | Durango, 243 m; 1.0418°N, 78.6240° W |
| QCAZ 46914 | *S. tsachila* | EC | Esmeraldas | San Francisco, 56 m; 1.0943°N, 78.7075° W |
| QCAZ 49641 | *S. tsachila* | EC | Azuay | Manta Real, Río Patul, 330 m; 2.5678° S, 79.3665° W |
| QCAZ 51404 | *S. tsachila* | EC | Imbabura | Lita-Balneario road, 600 m; 0.8630°N, 78.4531° W |
| QCAZ 51405 | *S. tsachila* | EC | Imbabura | Lita-Balneario road, 600 m; 0.8630°N, 78.4531° W |
| QCAZ 51881 | *S. tsachila* | EC | Esmeraldas | Durango, 202 m; 1.0427°N, 78.6244° W |
| QCAZ 53618 | *S. tsachila* | EC | Azuay | Recinto La López, Produmin S.A. mining camp, 425 m; 3.0873° S, 79.7149° W |
| QCAZ 55577 | *S. tsachila* | EC | Esmeraldas | Muisne-Cabo San Francisco, Laguna del Diablo, 49 m; 0.6544°N, 79.0488° W |
| QCAZ 56839 | *S. tsachila* | EC | Esmeraldas | Durango, 264 m; 1.0379°N, 78.6222° W |
| QCAZ 57045 | *S. tsachila* | EC | Esmeraldas | Tundaloma Lodge, 56 m; 1.1814°N, 78.7492° W |
| QCAZ 58029 | *S. tsachila* | EC | El Oro | Reserva Ecológica Buenaventura, 976 m; 3.6492° S, 79.7456° W |
| QCAZ 58046 | *S. tsachila* | EC | El Oro | Reserva Ecológica Buenaventura, 979 m; 3.6488° S, 79.7459° W |
| QCAZ 58047 | *S. tsachila* | EC | El Oro | Reserva Ecológica Buenaventura, 979 m; 3.6488° S, 79.7459° W |
| QCAZ 58048 | *S. tsachila* | EC | El Oro | Reserva Ecológica Buenaventura, 968 m; 3.6492° S, 79.7458° W |
| QCAZ 58049 | *S. tsachila* | EC | El Oro | Reserva Ecológica Buenaventura, 968 m; 3.6492° S, 79.7458° W |
| QCAZ 58649 | *S. tsachila* | EC | Esmeraldas | Durango, San Lorenzo road, km 37-38, 240 m; 1.0412°N, 78.6232° W |
| QCAZ 58652 | *S. tsachila* | EC | Esmeraldas | Durango, San Lorenzo road, Km 17, 23 m; 1.1652°N, 78.7527° W |
| QCAZ 58653 | *S. tsachila* | EC | Esmeraldas | Durango, San Lorenzo road, Km 17, 23 m; 1.1652°N, 78.7527° W |
| QCAZ 59056 | *S. tsachila* | EC | Los Ríos | Centro Cientifico Río Palenque, 168 m; 0.5965° S, 79.3615° W |
| QCAZ 60019 | *S. tsachila* | EC | Esmeraldas | La Unión, 96 m; 0.2185°N, 79.3533° W |
| QCAZ 61681 | *S. tsachila* | EC | Pichincha | Mindo, 1281 m; 0.0506° S, 78.7718° W |
| QCAZ 61682 | *S. tsachila* | EC | Pichincha | Mindo, 1281 m; 0.0506° S, 78.7718° W |
| QCAZ 61683 | *S. tsachila* | EC | Pichincha | Mindo, 1281 m; 0.0506° S, 78.7718° W |
| QCAZ 61684 | *S. tsachila* | EC | Pichincha | Mindo, 1281 m; 0.0506° S, 78.7718° W |
| QCAZ 61685 | *S. tsachila* | EC | Pichincha | Mindo, 1281 m; 0.0506° S, 78.7718° W |
| QCAZ 61686 | *S. tsachila* | EC | Pichincha | Mindo, 1281 m; 0.0506° S, 78.7718° W |
| QCAZ 61687 | *S. tsachila* | EC | Pichincha | Mindo, 1281 m; 0.0506° S, 78.7718° W |
| QCAZ 61688 | *S. tsachila* | EC | Pichincha | Mindo, 1281 m; 0.0506° S, 78.7718° W |
| QCAZ 61689 | *S. tsachila* | EC | Pichincha | Mindo, 1281 m; 0.0506° S, 78.7718° W |
| QCAZ 61940 | *S. tsachila* | EC | El Oro | La Cadena, 200 m; 3.2667° S, 79.7349° W |
| QCAZ 62535 | *S. tsachila* | EC | El Oro | Reserva Biológica Buenaventura, 975 m; 3.6491° S, 79.7458° W |
| QCAZ 62700 | *S. tsachila* | EC | Guayas | Balzar, 43 m; 1.1755° S, 79.9644° W |
| QCAZ 63070 | *S. tsachila* | EC | El Oro | Buenaventura, 1066 m; 3.6345° S, 79.7506° W |
| QCAZ 63503 | *S. tsachila* | EC | Pichincha | 1 km E Pedro Vicente Maldonado, 670 m; 0.0866°N, 79.0373° W |
| QCAZ 63504 | *S. tsachila* | EC | Pichincha | 1 km E Pedro Vicente Maldonado, 670 m; 0.0866°N, 79.0373° W |
| QCAZ 63505 | *S. tsachila* | EC | Pichincha | 1 km E Pedro Vicente Maldonado, 670 m; 0.0866°N, 79.0373° W |
| QCAZ 65690 | *S. tsachila* | EC | Esmeraldas | Reserva Tesoro Escondido, 264 m; 0.542°N, 79.1449° W |
| QCAZ 66641 | *S. tsachila* | EC | Esmeraldas | Durango, Tundaloma Lodge, Los Cucos trail, 59 m; 1.1829°N, 78.7527° W |
| QCAZ 66642 | *S. tsachila* | EC | Esmeraldas | Durango, Tundaloma Lodge, Los Cucos trail, 59 m; 1.1829°N, 78.7527° W |
| QCAZ 66643 | *S. tsachila* | EC | Esmeraldas | Durango, Tundaloma Lodge, Los Cucos trail, 59 m; 1.1829°N, 78.7527° W |
| QCAZ 66644 | *S. tsachila* | EC | Esmeraldas | Durango, 20 km SE Tundaloma Lodge, 265 m; 1.0873°N, 78.6220° W |
| QCAZ 66645 | *S. tsachila* | EC | Esmeraldas | Durango, Tundaloma Lodge, Los Cucos trail, 83 m; 1.1813°N, 78.7514° W |
| QCAZ 66646 | *S. tsachila* | EC | Esmeraldas | Durango, Tundaloma Lodge, Los Cucos trail, 59 m; 1.1829°N, 78.7527° W |
| QCAZ 66647 | *S. tsachila* | EC | Esmeraldas | Durango, Tundaloma Lodge, Los Cucos trail, 83 m; 1.1813°N, 78.7509° W |
| QCAZ 66648 | *S. tsachila* | EC | Esmeraldas | Durango, 20 km SE Tundaloma Lodge, 265 m; 1.0873°N, 78.6220° W |
| QCAZ 66650 | *S. tsachila* | EC | Esmeraldas | Durango, Tundaloma Lodge, Los Cucos trail, 59 m; 1.1829°N, 78.7527° W |
